# Metabolic side effects of antipsychotic drugs in individuals with schizophrenia during medium- to long-term treatment: protocol for a systematic review and network meta-analysis of randomized controlled trials

**DOI:** 10.1186/s13643-021-01760-z

**Published:** 2021-08-02

**Authors:** Johannes Schneider-Thoma, Angelika Kapfhammer, Dongfang Wang, Irene Bighelli, Spyridon Siafis, Hui Wu, Wulf-Peter Hansen, John M. Davis, Georgia Salanti, Stefan Leucht

**Affiliations:** 1 Department of Psychiatry and Psychotherapy, School of Medicine, Technical University of Munich, Ismaninger Straße 22, 81675 Munich, Germany; 2 Shanghai Key Laboratory of Psychotic Disorders, Shanghai Mental Health Center, Shanghai Jiao Tong University School of Medicine, Shanghai, China; 3 BASTA - das Bündnis für psychisch erkrankte Menschen , Munich, Germany; 4 Psychiatric Institute, University of Illinois at Chicago, Chicago, IL USA; 5 Department of Psychiatry, Johns Hopkins University , Baltimore, Maryland USA; 6 Institute of Social and Preventive Medicine, University of Bern, Bern, Switzerland

**Keywords:** Network meta-analysis, Antipsychotic drugs, Metabolic, Schizophrenia, Randomized controlled trials, Weight, Cholesterol, Triglyceride, Glucose

## Abstract

**Background:**

Antipsychotic drugs and especially the newer compounds are known to cause metabolic side effects. However, a comprehensive comparison of the different substances regarding their propensity to cause metabolic side effects in medium- to long-term treatment of schizophrenia is lacking.

**Methods:**

We will conduct a systematic review and network meta-analysis (NMA). We will include randomized controlled trials (RCTs) in which participants received either placebo or an antipsychotic (i.e. placebo-controlled trials and head-to-head comparisons of drugs). We will include studies in individuals with schizophrenia or related disorders (such as schizophreniform or schizoaffective disorders) at any stage of the disease (acute episode; maintenance phase). We will include studies with a duration of more than 3 months (medium- to long-term treatment). The primary outcome will be the change in body weight. Secondary outcomes will be the further metabolic parameters: fastening glucose, total cholesterol, low-density lipoprotein (LDL) cholesterol, high-density lipoprotein (HDL) cholesterol and triglycerides. We will search for eligible studies (independent of the publication status) in Cochrane Schizophrenia Group’s Study-Based Register of Trials, which is compiled by regular searches in trial registries and multiple electronic databases from their inception onwards including MEDLINE, EMBASE and PsycINFO. Additionally, we will search previously published systematic reviews and websites of pharmaceutical companies for eligible studies. At least two reviewers will independently conduct the process of study selection and data extraction. We will use the Cochrane Risk of Bias 2 tool to evaluate the risk of bias in studies. We will conduct random-effects NMA within a Bayesian framework to synthesize all evidence for each outcome. We will conduct sensitivity and subgroup analyses to assess the robustness of the findings and to explore heterogeneity. The confidence in the results will be evaluated using the Confidence in Network Meta-Analysis (CINeMA) framework.

**Discussion:**

This systematic review and network meta-analysis will provide a synthesis of the existing evidence from RCTs how antipsychotic drugs differ in terms of metabolic side effects during medium- to long-term treatment. The findings have the potential to influence the choice of antipsychotic medication made by individuals with schizophrenia and their physicians.

**Systematic review registration:**

PROSPERO CRD42020175414

**Supplementary Information:**

The online version contains supplementary material available at 10.1186/s13643-021-01760-z.

## Background

Schizophrenia is a debilitating and often life-long disorder that ranks among the 20 top causes of disability according to the Global Burden of Disease Study [1]. Antipsychotic drugs are the mainstay of the treatment of schizophrenia. They are proven to be effective for the acute phase [2] and for recurrence prophylaxis [3], and many individuals take antipsychotics for years, or even lifetime [4, 5]. However, antipsychotics cause important side effects, most importantly extrapyramidal motor disorders, increase in prolactin and metabolic side effects [6]. Metabolic side effects comprise weight gain, disturbances in cholesterol and triglyceride metabolism (dyslipidaemia) and dysregulation of glucose homeostasis (insulin resistance extending in diabetes) [7]. Obesity, dyslipidaemia and insulin resistance are key components of the metabolic syndrome [8–13] and are associated with important somatic diseases such as cardiovascular disorders, stroke and diabetes [14]. Thereby, metabolic side effects of antipsychotics are likely to contribute to the on average 14.5 years reduced life-span of individuals with schizophrenia [15]. Moreover, weight gain is an important subjective factor reducing the quality of life among persons with schizophrenia [16]. As a consequence, metabolic side effects are also linked to drug non-adherence [17], resulting in poor treatment outcomes and psychotic relapses. The decision of clinicians and individuals with schizophrenia which antipsychotic compound to use is often based on the profile of side effects because antipsychotic substances do not differ much in efficacy, but enormously in side effects [6]. Therefore, it is a field of big interest and ongoing research.

Recently Pillinger et al. [18] conducted a network meta-analysis focussing on the comparative effects of 18 antipsychotics on metabolic function during short-term treatment of patients with schizophrenia. The median treatment duration of the included trials was 6 weeks (IQR 6–8). However, it can be assumed that the metabolic side effects have not peaked after few weeks of treatment yet, and data of longer treatment duration could differ. Up to date, it is unknown how antipsychotic compounds differ in extent and time pattern of causing metabolic side effects in medium- to long-term use. Pérez-Iglesias et al. [19] observed changes in weight and lipids especially during the course of the first year (in a cohort of first episode patients treated with haloperidol, olanzapine and risperidone for up to 3 years). Millen et al. [20] showed in a pooled analysis of 86 clinical trials including adults treated with the oral or depot formulations of olanzapine that weight increases most rapidly in the first 3 months of treatment, and then the increase slows down but continues, tending to reach a plateau after 6–12 months. De Hert et al. [7] assumed that the greatest amount of weight gain associated with antipsychotic therapy in previously drug-naive individuals with schizophrenia occurs on a scale of few months. Thus, analysing the metabolic parameters after several months of treatment in an expected nearly “steady-state” can deliver important information on how antipsychotics differ in the medium- to long-term treatment. To date, evidence is still fragmentary, and there is no comprehensive comparison of this kind.

### Objective

The objective is to estimate relative propensities of antipsychotic drugs to cause metabolic side effects in medium- to long-term treatment of individuals with schizophrenia, in terms of the following:

1. Weight gain.
2. Disturbances in lipid and glucose metabolism.

## Methods

This systematic review and network meta-analysis is registered in the PROSPERO database (registration number: CRD42020175414). This protocol is being reported in accordance with the reporting guidance provided in the Preferred Reporting Items for Systematic Review and Meta-Analysis Protocols (PRISMA-P) statement [21] (see checklist in Additional file 1). In reporting the methods and results in the final report, we will follow the PRISMA 2020 statement [22] and the extension for reporting network meta-analysis of healthcare interventions [23].

### Criteria for considering studies for this review

#### Types of studies

Randomized controlled trials (RCTs) in which participants received either a placebo or an antipsychotic (i.e. placebo-controlled trials and head-to-head comparisons of drugs) will be included. Studies whose randomization process is at high risk of bias (according to Cochrane Risk of Bias 2 [24]) will be excluded.

We will accept open and blinded RCTs. We will include studies with a minimum duration of 3 months, which is medium- to long-term treatment according to the classification of the Cochrane Schizophrenia Group. In case of cross-over studies, we will use only the first cross-over phase in order to avoid the problem of carryover effects which are very likely in drugs for schizophrenia [25]. We will exclude cluster-randomized trials to avoid intransitivity compared to patient-randomized trials. There will be no language restriction to minimize “language bias” [26]. We will exclude studies from Mainland China because of serious quality concerns previously described in the literature [27–29]. To include the high number of Chinese studies without clarifying their study quality has the risk of introducing an important bias in the results. But like observed in a past project of ours [30], and also described by Tong et al. [28], it is difficult to contact Chinese authors and obtain information to clear out quality concerns. An exception is studies conducted in China by international drug companies that ensure high-quality standards.

#### Types of participants

Participants are individuals with a diagnosis of schizophrenia or related disorders (such as schizophreniform or schizoaffective disorders). There is no clear evidence that the latter schizophrenia-like psychoses are caused by fundamentally different disease processes or require different treatment approaches [31]. We will include trials irrespective of the diagnostic criteria used following the strategy of the Cochrane Schizophrenia Group [32]. This decision should increase generalizability and representativeness. Nevertheless, we will exclude studies that did not use operationalized criteria such as ICD-10 or DSM-IV in a sensitivity analysis.

There will be no restrictions in terms of gender, ethnicity, age or setting (inpatients or outpatients). Also, we will include individuals irrespective of the stage of the disease (acute episode; maintenance phase) because the occurrence of side effects can be considered largely independent of psychopathology at study start.

#### Interventions and comparators

We will include placebo and a broad collection of antipsychotic drugs, comprising all newer antipsychotics developed in the last decades (formerly called second-generation antipsychotics (SGAs)) and the clinically most important older antipsychotics (formerly called first-generation antipsychotics (FGAs)) (agents are listed in the “Search strategy” section). The choice of the latter was informed by a survey of international schizophrenia experts [33].

We will include all these compounds in any form of administration, oral or as intramuscular depot. If an antipsychotic is available in both, oral and depot form, both formulations will be used as separate interventions in the network, because there are indications that oral and depot applications can differ in propensity to cause side effects [34], e.g. due to pharmacokinetic or compliance issues [35]. Only short-acting intramuscular antipsychotics will be excluded because these are exclusively used in emergency situations nowadays. In fixed-dose studies, we will only include target to maximum doses according to the International Consensus Study on Antipsychotic Dosing [36]. All flexible-dose treatment regimens will be included, because these allow the investigators to titrate to the adequate dose for the individual study participant.

### Outcome measures

#### Primary outcome

Our primary outcome is weight gain measured in kilogrammes. Body weight is easy to measure and frequently used in clinical practice.

We will extract also other outcomes used to describe body weight, namely body mass index (BMI), waist circumference and number of study participants overweight, obese, with clinically significant weight gain (typically defined as weight gain ≥ 7% of baseline weight) and increased waist circumference. We plan to analyse these parameters as secondary outcomes only when sufficient data is available.

#### Secondary outcomes

Because reporting of metabolic side effects is not standardized in trials of schizophrenia, we will extract different parameters for the other domains of the metabolic syndrome (laboratory measures taken from blood samples) and plan to analyse for each domain the parameter with the most data available:

1. Glucose metabolism: levels of fastening glucose, glycated haemoglobin (HbA1c), homeostatic model assessment of insulin resistance (HOMA-IR) and insulin and number of study participants with impaired fastening glucose and increased HbA1c.
2. Disturbances in total cholesterol metabolism: total cholesterol and number of study participants with hypercholesterinaemia.
3. Disturbances in low-density lipoprotein (LDL) cholesterol metabolism: LDL cholesterol and number of study participants with increased LDL cholesterol.
4. Disturbances in high-density lipoprotein (HDL) cholesterol metabolism: HDL cholesterol and number of study participants with reduced HDL cholesterol.
5. Disturbances in triglyceride metabolism: triglycerides and number of study participants with hypertriglyceridaemia.

The pathological thresholds of these parameters (relevant for the dichotomous outcome measures) will be used as defined by the original study authors.

#### Time point of outcome measurement

We will analyse the outcomes at more than 3 months, which is medium/long-term according to the Cochrane Schizophrenia Group. The primary time point will be the study endpoint.

### Search strategy

#### Electronic searches

The literature search will be conducted in Cochrane Schizophrenia Group’s Study-Based Register of Trials with no date/time, language, document type and publication status limitations. This specialized register for clinical trials of interventions for schizophrenia is compiled of regular searches in multiple electronic databases including MEDLINE, EMBASE, Allied and Complementary Medicine (AMED), Cumulative Index to Nursing and Allied Health Literature (CINAHL), PsycINFO, PubMed, US National Institute of Health Ongoing Trials Register (ClinicalTrials.gov), World Health Organization International Clinical Trials Registry Platform (www.who.int/ictrp), ProQuest Dissertations and Theses A&I and its quarterly update, and Chinese databases (Chinese Biomedical Literature Database, China Knowledge Resource Integrated Database and Wanfang) and their annual updates until the end of 2016 [37–40].

Because of poor reporting of outcomes in medical research [41], we do not limit the search adding specific outcomes so that we will have all the outcomes. In the Intervention Field of STUDY, we will search for all references with the following antipsychotic compounds: (*Amisulpride* OR *Aripiprazole* OR *Asenapine* OR *Benperidol* OR *Brexpiprazole* OR *Cariprazine* OR *Chlorpromazine* OR *Clopenthixol* OR *Clozapine* OR *Flupentixol* OR *Fluphenazine* OR *Fluspirilene* OR *Haloperidol* OR *Iloperidone* OR *Levomepromazine* OR *Loxapine* OR *Lumateperone* OR *Lurasidone* OR *Molindone* OR *Olanzapine* OR *Paliperidone* OR *Penfluridol* OR *Perazine* OR *Perphenazine* OR *Pimozide* OR *Quetiapine* OR *Risperidone* OR *Sertindole* OR *Sulpiride* OR *Thioridazine* OR *Tiotixene* OR *Trifluoperazine* OR *Ziprasidone* OR *Zotepine* OR *Zuclopenthixol*).

A draft search for Cochrane Schizophrenia Group’s register is provided in Additional file 2 and the specific search strategies for the multiple electronic databases used to compile the register in Additional file 3.

#### Reference lists and other sources

We will check previously published relevant systematic reviews if the included studies met our inclusion criteria as well. For missing information, we will contact via email the corresponding authors and the responsible drug companies of each included study published in the last 20 years.

### Identification and selection of studies

We will list all studies identified through electronic and manual searches with citation, titles and abstracts in a reference management programme Citavi (Swiss Academic Software, Zurich, Switzerland). By inspecting all titles and abstracts, two authors will independently assess the eligibility of the potential studies identified in the literature searches. Disagreement will be resolved by discussion, and where doubt still remains, we will acquire the full article for further inspection. Once the full articles are obtained, at least two reviewers will independently decide whether the studies meet the inclusion criteria described above. If disagreement cannot be clarified by discussion, we will resolve it with a third reviewer or by consulting the study authors.

### Data extraction

At least two reviewers will independently extract data from each included trial on standardized and specifically customized digital forms in a Microsoft Access database. The software will automatically detect any inconsistencies in data extraction. Disagreement will be resolved by a discussion with a third reviewer or by seeking further information from the study authors.

We will collect the following data from each included study:

- Study characteristics: e.g. study citation, year of publication, sample size, diagnosis investigated and diagnostic criteria used, funding/sponsor and registration number to trial registries.
- Characteristics of study participants: e.g. age, gender, ethnicity, baseline metabolic parameters and lifetime exposure to antipsychotics (if not available, duration of illness will be used as a proxy).
- Intervention details: e.g. compound, application form, dosage and treatment duration.
- Outcome measures of the primary and secondary outcomes (see above), including information on imputation methods to handle missing data and the timing of recording the outcome (in weeks after randomization).

### Effect size measures

The effect size for continuous outcomes will be mean differences (MD) and its 95% credibility intervals (CrIs). For missing standard deviations (SDs), we will proceed as described in Sect. 6.5.2. of the *Cochrane Handbook for Systematic Reviews of Interventions* [42]. Change data will be preferred over endpoint data. For dichotomous outcomes, we will use odds ratio (OR) and its 95% CrIs.

### Handling of missing outcome data

Estimates based on imputation methods to handle missing data (used by the original authors) will be preferred over completers’ data. Imputed data based on mixed-models of repeated measurement (MMRM) or multiple imputations (MI) will be preferred over the last observation carried forward (LOCF), if available. For the primary outcome, we collect both, estimates based on imputation methods and completers’ data, to conduct the sensitivity analysis mentioned below.

### Risk of bias assessment

Two reviewers will independently assess the risk of bias for the included studies using the revised Cochrane Risk of Bias Tool (RoB) 2 [24]. The following domains will be checked:

1. Risk of bias arising from the randomization process.
2. Risk of bias due to deviations from the intended interventions (effect of assignment to intervention).
3. Risk of bias due to missing outcome data.
4. Risk of bias in the measurement of the outcome.
5. Risk of bias in the selection of the reported result.
6. Overall risk of bias, as calculated by the algorithm suggested within this tool.

When disagreement between the two independent reviewers arises, we will resolve it through discussion and, if needed, a third senior author will be involved. Where necessary, we will contact the authors of the original studies for further information. Effects of studies with a high risk of bias in the overall rating will be analysed by sensitivity analyses.

### Data analysis

#### Characteristics of the included studies

We will generate descriptive statistics of study characteristics (by study and overall), describing important clinical or methodological variables, such as age, gender, ethnicity, baseline metabolic parameters, lifetime exposure to antipsychotics (if not available, duration of illness will be used as a proxy), antipsychotic dose, blinding, use of enriched design, sponsorship and study duration.

#### Assessment of the transitivity assumption

Joint analysis of different treatment comparisons can only provide valid findings if the network is transitive. That means that trial participants who fulfil the inclusion criteria are equally likely to be randomized to any of the studies of interest (i.e. jointly randomizable) and that the observed comparisons do not differ with respect to the distribution of effect modifiers. We will assess this assumption epidemiologically by comparing the distribution of potential effect modifiers across studies grouped by comparison [43], namely age, sex, baseline weight, antipsychotic dose and study duration.

#### Assessment of heterogeneity

We will investigate heterogeneity (variability in relative treatment effects within the same treatment comparison) by visual inspection of forest plots and by estimating the between-study variance *τ*^2^ and with the *I*^2^ statistic. In the joint synthesis using NMA, we will assume the heterogeneity variance common across the various treatment comparisons and use empirical distributions to characterize the amount of heterogeneity as low, moderate or high using the first and third quantiles [44, 45]. We will explore potential reasons for heterogeneity by subgroup analysis (see below).

#### Network meta-analysis

Network meta-analysis (NMA) combines direct and indirect evidence for all relative treatment effects and can therefore provide estimates with maximum power and increased precision [46]. If the conditions for NMA are met (high likelihood of transitivity), we will perform random effects network meta-analyses using a mixture of frequentist (netmeta package for plotting the network and evaluate the inconsistency, as described below) and Bayesian methods (to fit the model and obtain all relative effects, using self-programmed routines and the rjags package) in the R software. A common heterogeneity parameter will be used in the model. Additionally, to assess how much the common heterogeneity affects the relative effect with respect to the extra uncertainty anticipated in a future study, we will estimate the prediction intervals.

A league table will present the summary MDs or ORs for all pairwise comparisons. If the estimation of the treatment effects is relatively precise, we will compute the probability for the ranking of each intervention using surface under the cumulative ranking curve (SUCRA) [47].

If the requirements of network meta-analysis are not met (low likelihood of transitivity and/or large unexplained inconsistency, see below) we will use pairwise meta-analysis for data synthesis. We will perform frequentist random effects pairwise meta-analysis in R using the meta package.

#### Geometry of the network

We will present the geometry of the networks by network plots. For each outcome, interventions which have been compared in clinical trials and for which data on the outcome (direct evidence) is available will be linked by lines. The thickness of lines will correspond to the number of trials evaluating the comparison. This size of circles around each intervention will correspond to the number of participants assigned to the intervention. Additionally, we will indicate the compared interventions of each study in a “characteristics of included studies”-table (see above) and the “risk of bias”-tables to display the characteristics of interventions and comparisons in the network.

#### Assessment of inconsistency

If transitivity is met, direct and indirect evidence should yield consistent results. We will check carefully the network for inconsistencies, because in a sample of medical network meta-analyses, about one-eighth of the networks were found to be inconsistent and leading to possible misinterpretation of results [48].

To evaluate consistency, local and global statistical methods will be used [49]. The design-by-treatment interaction test will examine the whole network [50], and the Separating Indirect from Direct Evidence (SIDE) approach will search for local inconsistencies [51]. Both will be fit using the netmeta package in R. In case of significant inconsistency, we will investigate the possible sources of it (mistakes in data entry, clear differences in study characteristics), and if necessary, we will explore the inconsistency further by network meta-regression and subgroup analyses (see below).

#### Exploring heterogeneity and inconsistency

We expect the presence of some heterogeneity and inconsistency. We will explore the following potential effect modifiers of the primary outcome by subgroup/meta-regression analyses:

1. Baseline weight.
2. Age.
3. Sex (percentage women).
4. Ethnicity.
5. Lifetime exposure to antipsychotics (if not available, duration of illness will be used as a proxy).
6. Antipsychotic dose.
7. Pharmaceutical sponsorship.
8. Time of recording the outcome since randomization.

#### Analyses to test the robustness of the results

We will perform the following sensitivity analyses on the primary outcome:

1. Exclusion of non-double-blind studies (open and single-blind studies).
2. Analysis of only data of observed cases.
3. Exclusion of studies that did not use operationalized criteria to diagnose schizophrenia.
4. Exclusion of studies with an overall assessment of high risk of bias.
5. Exclusion of studies in study participants with minimal prior exposure to antipsychotics, in particular, trials in first-episode patients and most studies in children.
6. Exclusion of enriched design studies. In enriched design studies, trial participants are first stabilized on one compound and then randomized to either staying on the same compound or switching to another compound.

#### Publication bias

We will explore the association between study size and effect size with a comparison-adjusted funnel plot and with a contour enhanced funnel plot of all active drugs versus placebo (when at least 10 studies are available per outcome) [52]. Any asymmetry observed can be attributed to systematic differences between small and large studies, true heterogeneity or publication bias.

#### Statistical software

The analysis and presentation of results will be performed using self-programmed routines and the rjags, meta and netmeta package in R (details see above).

#### Assessment of the confidence in the evidence from NMA

We will evaluate the confidence in estimates of the primary outcome with the framework Confidence in Network Meta-Analysis (CINeMA) [53, 54], implemented in a web application [55].

## Discussion

This network meta-analysis will examine the differences in metabolic side effects (comprising weight gain and disturbances in lipid and glucose metabolisms) of antipsychotic drugs during medium- to long-term treatment of individuals with schizophrenia.

We anticipate the following practical or operational issues involved in performing the review: First, we expect several hundreds of included studies for which data needs to be extracted in double. To handle this enormous amount of information, we are planning to use a Microsoft Access database customized for this purpose allowing automatic comparison of data entries. Second, metabolic side effects will not have been systematically recorded in many, especially older, trials and may appear only in adverse event listings, which are typically reported incompletely in publications. Also in modern trials, reporting of metabolic parameters is not standardized and may be incomplete. Thus, we are planning to contact pharmaceutical companies and study authors to request for missing data. Third, a related and frequent problem is missing measures of variability of effect, i.e. standard deviations (SDs). In this case, we will calculate them from other reported statistical parameters as recommended by the *Cochrane Handbook for Systematic Reviews of Interventions* [42] or request missing SDs from the study authors. When no information can be obtained, we will derive SDs from those of the other studies using a validated imputation technique [56]. Fourth, several trials will investigate different dose arms of the same drug. For the primary analysis, different doses (when within the recommended range [36]) will be pooled, but the effects of the dose will be explored in subgroup analysis.

Possible limitations at individual study and review levels may arise from the following issues: The above-mentioned problem of missing data could potentially result in publication bias. We will assess this risk and include it in the CINeMA assessment. Another limitation at the study level is premature study discontinuations, which are frequent in trials with participants with schizophrenia and which increase with longer study duration. Imputation methods used by the original authors aim to correct this issue but cannot fully overcome this problem. To investigate whether premature study discontinuations substantially impact the relative treatment effects (resulting in a limitation at the review level), we are planning a sensitivity analysis using completer data only. Moreover, we will extract premature discontinuations overall and for reasons related to the outcomes and use this information to judge the risk of bias due to missing outcome data [24]; also, we will clearly state this issue in the limitation section. Additionally, differences between comparisons in population characteristics (such as baseline weight, age, sex, ethnicity and previous exposure to antipsychotics), intervention characteristics (such as dose) and study characteristics (such as blinding, diagnostic criteria, risk of bias, enriched design, pharmaceutical sponsorship and trial duration) may modify treatment effects and can be a limitation at the review level. Therefore, we will include these characteristics in the transitivity assessment and explore their impact on the results in meta-regression and sensitivity analyses. Of note, the remaining differences that result in imprecise, heterogeneous and/or inconsistent treatment effects in the NMA will lead to lower confidence in the estimate in CINeMA.

If any protocol amendments, e.g. for additional analyses to explore potential effect modifiers, will be needed, we will update the protocol record in PROSPERO and we will clearly state the changes to the protocol and their reasons in the resulting publication.

This network meta-analysis will summarize all available data from randomized controlled clinical trials to date and integrate direct evidence and indirect evidence. Thus, it can provide a comprehensive overview of the evidence with increased statistical power (by combining direct and indirect evidence) and with estimates available for comparisons of drugs that have not been compared in clinical trials so far (by using indirect evidence). It shall inform the choice of antipsychotic medication by individuals with schizophrenia and their physicians, thereby the side effect burden might be reduced and adherence to treatment increased which could improve the long-term outcome of this severe mental disorder.

## Supplementary Information

**Additional file 1.** PRISMA-P 2015 Checklist.

**Additional file 2.** Search strategy for the Cochrane Schizophrenia Group’s Study-Based Register of Trials.

**Additional file 3.** Specific search strategies for electronic databases used to compile the Cochrane Schizophrenia Group’s Study-Based Register of Trials.

## Data Availability

Not applicable.

## Abbreviations

CINeMA: Confidence in Network Meta-Analysis
CrI: Credibility interval
DSM: Diagnostic and Statistical Manual of Mental Disorders
ICD: International Classification of Diseases
IQR: Interquartile range
MD: Mean difference
NMA: Network meta-analysis
OR: Odds ratio
PRISMA-P: Preferred Reporting Items for Systematic Reviews and Meta-Analyses for Systematic Review Protocols
PROSPERO: International Prospective Register of Systematic Reviews
RCT: Randomized controlled trial
SD: Standard deviation
