# Supplementary material for: Metabolic side effects of antipsychotic drugs in individuals with schizophrenia during medium- to long-term treatment: protocol for a systematic review and network meta-analysis of randomized controlled trials
Source: Syst Rev. 2021 Aug 2;10:214. doi: 10.1186/s13643-021-01760-z (PMC8330017; doi:10.1186/s13643-021-01760-z)
Supplement: Supplementary file 2 — Additional file 2. Search strategy for the Cochrane Schizophrenia Group’s Study-Based Register of Trials. [file 13643_2021_1760_MOESM2_ESM.pdf]

Cochrane Schizophrenia Group's Study-Based Register of Trials

The information specialist will search the register using the following search strategy which has been developed based on literature review, consulting with the contact author of the review, and checking the indexed interventions in the Group's Register:

(*Amisulpride* OR *Aripiprazole* OR *Asenapine* OR *Benperidol* OR *Brexpiprazole* OR *Cariprazine* OR *Chlorpromazine* OR *Clopenthixol* OR *Clozapine* OR *Flupentixol* OR *Fluphenazine* OR *Fluspirilene* OR *Haloperidol* OR *Iloperidone* OR *Levomepromazine* OR *Loxapine* OR *Lumateperone* OR *Lurasidone* OR *Molindone* OR *Olanzapine* OR *Paliperidone* OR *Penfluridol* OR *Perazine* OR *Perphenazine* OR *Pimozide* OR *Quetiapine* OR *Risperidone* OR *Sertindole* OR *Sulpiride* OR *Thioridazine* OR *Tiotixene* OR *Trifluoperazine* OR *Ziprasidone* OR *Zotepine* OR *Zuclopenthixol*) in Intervention Field of STUDY

In such a study-based register, searching the major concept retrieves all the synonyms and relevant studies. This is because the studies have already been organised, based on their interventions, and linked to the relevant topics. This allows rapid and accurate searches that reduce waste in the next steps of systematic reviewing. The details of classification of pharmacological interventions and this register were reported elsewhere [1-4].

Following the methods from Cochrane [5], the Information Specialist compiles this register from systematic searches of major resources and their monthly updates (unless otherwise specified):

1. MEDLINE;
2. Embase;
3. Allied and Complementary Medicine (AMED);
4. Cumulative Index to Nursing and Allied Health Literature (CINAHL);
5. PsycINFO;
6. PubMed;
7. US National Institute of Health Ongoing Trials Register ClinicalTrials.gov;
8. World Health Organization International Clinical Trials Registry Platform (www.who.int/ictrp);

9. ProQuest Dissertations and Theses A&I and its quarterly update;
10. Chinese databases (Chinese Biomedical Literature Database, China Knowledge Resource Integrated Database, and Wanfang) and their annual updates until the end of 2016.

The register also includes handsearches and conference proceedings (see Group's website: http://schizophrenia.cochrane.org/register-trials). It does not place any limitations on language, date, document type or publication status.

References

1. Shokraneh F, Adams CE. Study-based registers of randomized controlled trials: Starting a systematic review with data extraction or meta-analysis. ***BioImpacts*** 2017;7(4):209-17. [DOI: 10.15171/bi.2017.25]
2. Shokraneh F, Adams CE. Study-based registers reduce waste in systematic reviewing: discussion and case report. ***Systematic Reviews*** 2019;8:129. [DOI: 10.1186/s13643-019-1035-3]
3. Shokraneh F, Adams CE. Classification of all pharmacological interventions tested in trials relevant to people with schizophrenia: A study-based analysis. ***Health Information and Libraries Journal*** [Accepted]
4. Shokraneh F, Adams CE. Cochrane Schizophrenia Group's Study-Based Register of Randomized Controlled Trials: Development and Content Analysis. ***Schizophrenia Bulletin Open*** 2020 [Revised]
5. Lefebvre C, Glanville J, Briscoe S, Littlewood A, Marshall C, Metzendorf M, et al. Searching for and selecting studies. In: Higgins JP, Thomas J, Chandler J, Cumpston M, Li T, Page MJ, Welch VA, editor(s). **Cochrane Handbook for Systematic Reviews of Interventions**. 2nd edition. John Wiley and Sons, 2019:67-107. [DOI: 10.1002/9781119536604.ch4]
