# Supplementary material for: Metabolic side effects of antipsychotic drugs in individuals with schizophrenia during medium- to long-term treatment: protocol for a systematic review and network meta-analysis of randomized controlled trials
Source: Syst Rev. 2021 Aug 2;10:214. doi: 10.1186/s13643-021-01760-z (PMC8330017; doi:10.1186/s13643-021-01760-z)
Supplement: Supplementary file 3 — Additional file 3. Specific search strategies for electronic databases used to compile the Cochrane Schizophrenia Group’s Study-Based Register of Trials. [file 13643_2021_1760_MOESM3_ESM.pdf]

List of Searches Resources
ABS Database
Host: PC-TM Ltd.
URL: http://nt-cmb.medun.acad.bg/absw/abs.htm
Geographical Coverage: Bulgaria
Dates Last Searched: 2004 [We need volunteers to update this search]
Time Coverage: Unclear – Present
Search Strategy:
Search option: Всички думи [all words]
#1 андом%
#2 двоино%
#3 сляпо%
#4 (клиничен% AND опит%)
#5 опит%
#6 random%
#7 alloc%
#8 assign%
#9 double-blind%
#10 placebo%
#11 (clinical% AND trial%)
#12 trial%
#13 or/1-12

AFRIMS: Armed Forces Research Institute for Medical Sciences
URL: http://www.afrims.org/frmsetlibrary.html
Geographical Coverage: Thailand
Dates Last Searched: February 11, 2015 [Planned Annual Update]
Time Coverage: 1960 – Present
Search Strategy:
#1 rando$
#2 double and blin$
#3 blin$
#4 #2 and #3
#5 #1 and #4

AFROLIB: World Health Organization Regional Office for Africa
URL: http://afrolib.afro.who.int/cgi-bin/wxis.exe/iah/?IsisScript=iah/iah.xic&lang=I&base=afrolib
Geographical Coverage: African Countries
Dates Last Searched: February 11, 2015 [Planned Annual Update]
Time Coverage: Unclear – Present
Search Strategy:
random OR randomized OR randomised OR randomly OR randomisation OR randomization
blind OR blinding
trial OR trials
placebo OR placebos

AIM (African Index Medicus): World Health Organization Regional Office for Africa
URL: http://indexmedicus.afro.who.int/cgi-bin/wxis.exe/iah/?IsisScript=iah/iah.xis&lang=I&base=AIM
Geographical Coverage: Africa
Dates Last Searched: February 11, 2015 [Planned Annual Update]
Time Coverage: Unclear – Present
Search Strategy:
random OR randomized OR randomised OR randomly OR randomisation OR randomization
blind OR blinding

trial OR trials
placebo OR placebos

AL MANHAL
URL: http://www.almanhal.com
Geographical Coverage: Arab and Islamic World
Dates Last Searched: February 11, 2015 [Planned Annual Update]
Time Coverage: Unclear – Present
Search Strategy:
schizophrenia [all subjects]
schizoaffective [all subjects]
psychosis [all subjects]

AMED: Allied and Complementary Medicine Database
Host: Ovid SP
URL: http://ovidsp.ovid.com/
Geographical Coverage: UK
Dates Last Searched: October 22, 2015 [Regular Monthly Updates]
Time Coverage: 1985 – Present
Search Strategy:
1. exp clinical trials/
2. exp randomized controlled trials/
3. exp double-blind method/
4. randomized controlled trial.pt.
5. clinical trial.pt.
6. controlled clinical trial.pt.
7. (clinic$ adj4 trial$).mp.
8. (random$ adj5 (assign$ or allocat$ or assort$)).mp.
9. (randomi$ adj5 control$ adj5 trial$).mp.
10. (crossover or cross-over).mp.
11. ((singl$ or doubl$ or trebl$ or tripl$) adj (blind$ or mask$)).mp.
12. exp random allocation/
13. or/1-12
14. exp Dyskinesia, Drug-Induced/
15. exp Movement Disorders/
16. exp psychotic disorders/
17. exp schizophrenia/
18. schizo$.mp.
19. hebephreni$.mp.
20. hebephreni$.mp.
21. oligophreni$.mp.
22. psychotic$.mp.
23. psychos#s.mp.
24. ((chronic$ or sever$) adj2 mental$ adj2 (ill$ or disorder$)).mp.
25. (tardiv$ adj dyskine$).mp.
26. akathisi$.mp.
27. (neuroleptic adj5 malignant adj2 syndrome).mp.
28. (movement adj5 (disorder or disorders)).mp.
29. neuroleptic-induc$.mp.
30. parkinsoni$.mp.
31. exp Parkinson Disease/
32. (parkinson?s adj disease).ti.
33. or/31-32
34. 30 not 33
35. or/14-29

36. 35 or 34
37. 13 and 36

ANZCTR: Australian New Zealand Clinical Trials Registry (ANZCTR)
Host: WHO ICTRCP
URL: http://www.who.int/trialsearch/
Geographical Coverage: Australia and New Zealand
Dates Last Searched: October 22, 2015 [Regular Monthly Updates]
Time Coverage: 2009 – Present
Search Strategy:
See WHO ICTRP

BiblioMedica: Aggregate collection of relevant Czech National Medical Library databases
URL: http://www.medvik.cz/bmc/
Geographical Coverage: Czech
Dates Last Searched: February 11, 2015 [Planned Annual Update]
Time Coverage: Unclear – Present
Search Strategy:
3. 1 and 2
2. "schiz*" OR psychosis OR "psychotic*" OR tardive OR akathisia OR parkinsonism OR "delusion*"
1. "random*" OR "placebo*" OR "trial*" OR "blind*"

Biological Abstracts
Host: Web of Knowledge
URL: http://isiknowledge.com/
Geographical Coverage: Multi-National [Focused on English-Speaking World]
Dates Last Searched: October 22, 2015 [via BIOSIS Previews; Regular Monthly Updates]
Time Coverage: 1925 – Present
Note: The combination of Biological Abstracts/RRM and Biological Abstracts is BIOSIS Previews.
Search Strategy:
#1 CLIN*
#2 TRIAL*
#3 #1 near #2
#4 SINGL*
#5 DOUBL*
#6 TREBL*
#7 TRIPL*
#8 BLIND*
#9 MASK*
#10 (#4 or #5 or #6 or #7) near (#8 or #9)
#11 RANDOMI*
#12 RANDOM*
#13 ALLOCAT*
#14 ASSIGN*
#15 #12 near (#13 or #14)
#16 CROSSOVER
#17 #16 or #15 or #11 or #10 or #3
#18 SCHIZO*
#19 HEBEPHRENI*
#20 OLIGOPHRENI*
#21 PSYCHOTIC*
#22 PSYCHOSIS
#23 PSYCHOSES
#24 CHRONIC*

#25 SEVER*
#26 MENTAL*
#27 ILL*
#28 DISORDER*
#29 ((CHRONIC* or SEVER*) near2 MENTAL*) near2 (ILL* or DISORDER*)
#30 #18 or #19 or #20 or #21 or #22 or #23 or #24
#31 TARDIV*
#32 DYSKINE*
#33 TARDIV* near DYSKINE*
#34 AKATHISI*
#35 ACATHISI*
#36 NEUROLEPTIC*
#37 MALIGNANT
#38 SYNDROME
#39 NEUROLEPTIC* and (MALIGNANT near2 SYNDROME)
#40 MOVEMENT
#41 DISORDER*
#42 #36 and MOVEMENT and DISORDER*
#43 PARKINSONI*
#44 NEUROLEPTIC-INDUC*
#45 PARKINSON'S
#46 DISEASE
#47 PARKINSON'S near1 (DISEASE in TI)
#48 #33 or #34 or #35 or #39 or #42 or #43 or #44
#49 #48 not #47
#50 #49 or #30

BIOSIS Previews
Host: Web of Knowledge
URL: http://isiknowledge.com/
Geographical Coverage: Multi-National [Focused on English-Speaking World]
Dates Last Searched: October 22, 2015 [Regular Monthly Updates]
Time Coverage: 1969 – Present
Search Strategy:
1 TS=schizo* OR TI=schizo* OR DS=schizo*
2 TS=psychosis* or TI=psychosis* OR DS=psychosis*
3 TS=psychotic* or TI=psychotic* OR DS=psychotic*
4 TS=psychoses* or TI=psychoses* OR DS=psychoses*
5 TS=oligophreni* or TI=oligophreni* OR DS=oligophreni*
6 TS=hebephreni* or TI=hebephreni* OR DS=hebephreni*
7 TS=tardiv* OR TI=tardiv* OR DS=tardiv*
8 TS=dyskine* OR TI=dyskine* OR DS=dyskine*
9 #8 AND #7
10 TS=akathisi* OR TI=akathisi* OR DS=akathisi*
11 TS=acathisi* OR TI=acathisi* OR DS=acathisi*
12 TS=neuroleptic malignant syndrome* OR TI=neuroleptic malignant syndrome* OR DS=neuroleptic malignant syndrome*
13 TS=neuroleptic movement disorder* OR TI=neuroleptic movement disorder* OR DS=neuroleptic movement disorder*
14 TS=severe mental illness* OR TI=severe mental illness* OR DS=severe mental illness*
15 TS=chronic mental illness* OR TI=chronic mental illness* OR DS=chronic mental illness*
16 TS=chronic mental disorder* OR TI=chronic mental disorder* OR DS=chronic mental disorder*
17 TS=severe mental disorder* OR TI=severe mental disorder* or DS=severe mental disorder*
18 TS=neuroleptic-induc* OR TI=neuroleptic-induc* OR DS=neuroleptic-induc*
19 DS=parkinson's disease

20 #18 OR #17 OR #16 OR #15 OR #14 OR #13 OR #12 OR #11 OR #10 OR #9 OR #6 OR #5 OR #4 OR #3 OR #2 OR #1
21 #20 NOT #19
22 MQ=Randomized clinical trial* OR DS=Randomized clinical trial* OR TS=Randomized clinical trial* OR TI=Randomized clinical trial*
23 DS=randomi* OR TI=randomi* OR TS=randomi*
24 DS=clin* OR TI=clin* OR TS=clin*
25 DS=trial* OR TS=trial* OR TI=trial*
26 #25 AND #24
27 DS=(singl* OR Doubl* OR Tripl* OR Trebl*) OR TS=(singl* OR Doubl* OR Tripl* OR Trebl*) OR TI=(singl* OR Doubl* OR Tripl* OR Trebl*)
28 DS=(mask* OR blind*) OR TS=(mask* OR blind*) OR TI=(mask* OR blind*)
29 DS=crossover* OR TS=crossover* OR TI=crossover*
30 DS=(allocate* OR assign*) OR TS=(allocate* OR assign*) OR TI=(allocate* OR assign*)
31 DS=random* OR TS=random* OR TI=random*
32 #31 AND #30
33 #32 OR #29 OR #28 OR #27 OR #26 OR #23 OR #22
34 #33 AND #21

CAJ: China Academic Journals
Host: East View Information Services
URL: http://online.eastview.com/login_china/index.jsp
Geographical Coverage: China
Dates Last Searched: January 31, 2015 [Regular Annual Updates]
Time Coverage: 2004 – Present
Search Strategy:
#1 Title: 精神分裂 and’ 随机
#2 Keyword: 精神分裂 and’ 随机
#3 Abstract: 精神分裂 and’ 随机

CBM: Chinese Biomedical Literature Database
Host: Chongqing VIP Information Co.,Ltd
URL: http://en.cqvip.com/
Geographical Coverage: China
Dates Last Searched: January 31, 2015 [Regular Annual Updates]
Time Coverage: 1978 - Present
Search Strategy:
精神分裂 and’ 随机

Chiangmai University E-Research: **ฐานข้อมูลงานวิจัยอิเล็กทรอนิกส์**
URL: http://library.cmu.ac.th/digital_collection/eresearch/
Geographical Coverage: Thailand
Dates Last Searched: November 2008 [We need volunteers to update this search]
Time Coverage: Unclear – Present
Search Strategy:
Search option: In all fields
#1 randomi*
#2 double blind
#3 สุ่ม
#4 #1 or #2 or #3

Chiangmai University E-thesis: **ฐานข้อมูลวิทยานิพนธ์อิเล็กทรอนิกส์**
URL: http://library.cmu.ac.th/digital_collection/etheses/index.php
Geographical Coverage: Thailand

Dates Last Searched: November 2008 [We need volunteers to update this search]
Time Coverage: Unclear – Present
Search Strategy:
#1 randomi*
#2 double blind
#3 สุ่ม
#4 #1 or #2 or #3

China VIP
URL: http://www.cqvip.com
Geographical Coverage: China
Dates Last Searched: January 31, 2015 [Regular Annual Updates]
Time Coverage: 1989 – Present
Search Strategy:
精神分裂 and' 随机

China Wanfang
URL: http://www.wanfangdata.com
Geographical Coverage: China
Dates Last Searched: January 31, 2015 [Regular Annual Updates]
Time Coverage: Unclear – Present
Search Strategy:
精神分裂 and' 随机

Chulalongkorn University: Chulalongkorn University Library Information Network
URL: http://library.car.chula.ac.th/
Geographical Coverage: Thailand
Dates Last Searched: February 11, 2015 [Planned Annual Update]
Time Coverage: Unclear – Present
Search Strategy:
Search option: medical
#1 randomi**
#2 double*
#3 blind*
#4 #2 and #3
#5 random*
#6 allo*
#7 assign*
#8 #6 or #7
#9 #5 and #8
#10 สุ่ม
#11 เปรียบเทียบ
#12 #1 or #4 or #9 or #10 or #11

CINAHL: Current Index to Nursing and Allied Health Literature A
Host: Ovid SP
Geographical Coverage: Multi-National
Dates Last Searched: 2006
Time Coverage: 1982 – Present
Note: This database is no longer available via Ovid SP and only is available via EBSCOhost.
Search Strategy:
1. pilot studies/
2. reproducibility of results/
3. exp clinical research/

4. exp clinical trials/
5. research methodology/
6. meta analysis/
7. crossover design/
8. patient selection/
9. random assignment/
10. sample size/
11. placebos/
12. comparative studies/
13. study design/
14. experimental studies/
15. community trials/
16. exp random sample/
17. research/
18. exp professional practice, research-based/
19. exp "outcomes (health care)"/
20. (research or clinical trial$).pt.
21. (practice guideline$ or systematic review$).pt.
22. nursing interventions.pt.
23. (Critical path or care plan or protocol).pt.
24. random$.tw.
25. (Efficacy or effectiveness).tw.
26. ((double or single or triple) adj blind$).tw.
27. placebo$.tw.
28. (sham or mask$).tw.
29. intention to treat.tw.
30. (control$ adj2 trial$).tw.
31. or/1-30

CINAHL: Current Index to Nursing and Allied Health Literature B
Host: EBSCO
URL: http://search.ebscohost.com/
Geographical Coverage: Multi-National
Dates Last Searched: October 22, 2015 [Regular Monthly Updates]
Time Coverage: 1982 – Present
Search Strategy:
S43 S41 and S42
S42 S37 or S38 or S39 or S40
S41 S1 or S2 or S3 or S4 or S5 or S14 or S15 or S16 or S19 or S20 or S21 or S22 or S26 or S30 or S31 or S32 or S33 or S34 or S35 or S36
S40 MW random* or TI random* or AB random* or IN random*
S39 MH triple-blind studies
S38 MH double-blind studies
S37 MH single-blind studies
S36 (MH "Neuroleptic Malignant Syndrome")
S35 (MH "Dyskinesia, Drug-Induced")
S34 (MH "Akathisia, Drug-Induced")
S33 (MH "Movement Disorders+")
S32 MW neuroleptic-induc* or TI neuroleptic-induc* or AB neuroleptic-induc* or IN neuroleptic-induc*
S31 MW parkinsoni* or TI parkinsoni* or AB parkinsoni* or IN parkinsoni*
S30 S22 and S29
S29 S27 and S28
S28 MW disorder or TI disorder or AB disorder or IN disorder
S27 MW movement or TI movement or AB movement or IN movement

S26 S22 and S25
S25 S23 and S24
S24 MW syndrome* or TI syndrome* or AB syndrome* or IN syndrome*
S23 MW malignant* or TI malignant* or AB malignant* or IN malignant*
S22 MW neuroleptic* or TI neuroleptic* or AB neuroleptic* or IN neuroleptic*
S21 MW acathisi* or TI acathisi* or AB acathisi* or IN acathisi*
S20 MW akathisi* or TI akathisi* or AB akathisi* or IN akathisi*
S19 S17 and S18
S18 MW dyskine* or TI dyskine** or AB dyskine* or IN dyskine*
S17 MW tardiv* or TI tardiv* or AB tardiv* or IN tardiv*
S16 MW hebephreni* or TI hebephreni* or AB hebephreni* or IN hebephreni*
S15 MW oligophreni* or TI oligophreni* or AB oligophreni* or IN oligophreni*
S14 S11 and S13
S13 S8 or S12
S12 S9 or S10
S11 S6 or S7
S10 MW disorder* or TI disorder* or AB disorder* or IN disorder*
S9 MW ill* or TI ill* or AB ill* or IN ill*
S8 MW mental* or TI mental* or AB mental* or IN mental*
S7 MW sever* or TI sever* or AB sever* or IN sever*
S6 MW chronic* or TI chronic* or AB chronic* or IN chronic*
S5 MW psychoses* or TI psychoses* or AB psychoses* or IN psychoses*
S4 MW psychotic* or TI psychotic* or AB psychotic* or IN psychotic*
S3 MW psychosis or TI psychosis or AB psychosis or IN psychosis
S2 MW schizo* or TI schizo* or AB schizo* or IN schizo*
S1 (MH "Psychotic Disorders+")

ClinicalStudyResults.org
URL: http://www.clinicalstudyresults.org/home/
Geographical Coverage: USA
Dates Last Searched: May 2011; February 11, 2015 [Inactive]
Time Coverage: Aug 2009 – May 2011
Search Strategy:
Search option: Studied indications or disease
#1 Schizophrenia
#2 Schizophrenia (adolescents)
#3 Schizophrenia consta
#4 Schizophrenia maintenance
#5 Schizophrenia & schizoaffective disorder
#6 Schizophrenia acute exacerbation
#7 Schizophrenia bipolar disorder & psychotic disorder nos
#8 Schizophrenia treatment resistant
#9 Acute Schizophrenia
#10 Agitation
#11 Long-acting injection, Schizophrenia
#12 Mania
#13 Psychosis
#14 Psychotic disorders
#15 Schizoaffective disorder, schizophreniaform disorder

ClinicalTrials.gov
URL: http://clinicaltrials.gov/
Geographical Coverage: Multi-National
Dates Last Searched: October 22, 2015 [Regular Monthly Updates]
Time Coverage: 2000 – Present

Search Strategy:
Tardive dyskinesia OR schizophrenia OR schizophreniform OR schizoaffective OR psychosis OR akathisia
Limit to interventional studies

Counseling and Psychotherapy: Counseling and Psychotherapy Transcripts, Client Narratives, and Reference Works
URL: http://asp6new.alexanderstreet.com/psyc/psyc.index.map.aspx
Geographical Coverage: USA
Dates Last Searched: July 14, 2009; October 22, 2015 [Inactive]
Time Coverage: Unknown
Search Strategy:
Search option:
Full text
Material Type=Session Transcript OR Client Narrative OR Reference OR Editorial
#1 randomi*
#2 schizo*
#3 #1 AND #2

CRISP: Computer Retrieval of Information on Scientific Projects
URL: https://projectreporter.nih.gov/reporter.cfm
Geographical Coverage: USA
Dates Last Searched: February 11, 2015 [Planned Annual Update]
Time Coverage: Unclear – Present
Note: CRISP has been replaced by RePORT Expenditures and Results (RePORTER)
Search Strategy:
Search option: ALL in other fields except research grants or projects
#1 schizophrenia
#2 randomized
#3 randomly
#4 #2 or #3
#5 #1 and #4

CSA: CSA Sociological Abstracts
Host: ProQuest
URL: http://search.proquest.com/
Geographical Coverage: Multi-National
Dates Last Searched: May 2007
Time Coverage: 1952 – Present
Note: CSA has been merged with ProQuest.
Search Strategy:
#1 psychoses
#2 catatonia
#3 chronic schizophrenia
#4 #1 or #2 or #3
#5 paranoid schizophrenia
#6 paraphrenia
#7 schizophreniform disorder
#8 parkinsoni*
#9 neuroleptic-induc*
#10 #8 and #9
#11 neuroleptic*
#12 movement
#13 disorder*
#14 #11 and #12 and #13

#15 malignant
#16 #11 and #15
#17 syndrome
#18 #16 within 2 #17
#19 chronic*
#20 sever*
#21 #19 or #20
#22 mental*
#23 #21 within 2 #22
#24 tardiv*
#25 dyskine*
#26 #24 within 2 #25
#27 ill*
#28 disorder*
#29 schizo*
#30 hebephreni*
#31 oligophreni*
#32 psychotic*
#33 psychosis
#34 psychoses
#35 #4 or #5 or #6 or #7 or #10 or #14 or #18 or #23 or #26 or #27 or #28 or #29 or #30 or #31 or #32 or #33 or #34
#36 random*
#37 randomi?ed controlled trials
#38 double* blind*
#39 double* blind*
#40 double blind studies
#41 single* near blind*
#42 #36 or #37 or #38 or #39 or #40 or #41
#43 #35 and #42

Current Controlled Trials (Now ISRCTN)
URL: http://www.isrctn.com/
Geographical Coverage: USA and Europe
Dates Last Searched: October 22, 2015 [Regular Monthly Updates]
Time Coverage: 2000 – Present
Search Strategy:
#1 schizo%
#2 psychosis
#3 psychoses
#4 psychotic
#5 tardive dyskinesia
#6 akathisia
#7 #1 or #2 or #3 or #4 or #5 or #6
Search Strategy Since 2015:
Schizophrenia
Schizotypal
Schizoaffective
Schizophreniform
Psychosis
Psychoses
Psychotic
Tardive Dyskinesia
Akathisia

Datastar PASCAL
Host: Datastar [Now ProQuest Dialog]
URL: http://www.datastarweb.com
Geographical Coverage: Europe
Dates Last Searched: May 20, 2005
Time Coverage: 1973 – Present
Note: The hosts for this database are ProQuest Dialog, Ovid SP, EBSCOhost and STN International.
Search Strategy:
#1 randomi$
#2 trial$
#3 stud$
#4 etud$
#5 estud$
#6 juicio$
#7 #2 or #3 or #4 or #5 or #6
#8 #1 same #7
#9 randomized-allocation
#10 randomized-clinical-trial
#11 randomized-control
#12 randomized-control-trial
#13 randomized-controlled-trial
#14 rct-randomized-controlled-trials
#15 randomized-design
#16 randomized-evaluation
#17 randomized-experiment
#18 randomized-protocol
#19 randomized-study
#20 non-randomized-study-design
#21 randomized-trial
#22 randomizedcontrolled
#23 randomizedmulticenter
#24 randomizedparallel
#25 randomizedplacebo
#26 randomizedstudies
#27 randomizedto)
#28 #8 or #9 or #10 #11 or #12 or #13 or #14 or #15 or #16 or #17 or #18 or #19 or #20 or #21 or #22 or #23 or #24 or #25 or #26 or #27
#29 crossover$
#30 placebo$
#31 aleatorizad$
#32 double$
#33 single$
#34 triple$
#35 #32 or #33 or #34
#36 blind$
#37 #36 near #35
#38 insu
#39 #32 near #38
#40 doble
#41 ciego
#42 #40 near #41
#43 par chance$
#44 par hazard$
#45 #28 or #29 or #30 or #31 or #37 or #39 or #42 or #43 or #44

Department of Mental Health Research
URL: http://www.dmh.go.th/
Geographical Coverage: Thailand
Dates Last Searched: 2008 [We need volunteers to update this search]
Time Coverage: Unclear – Present
Search Strategy:
#1 randomized
#2 randomised
#3 double-blind
#4 สุ่ม
#5 #1 or #2 or #3 or #4

EDINA BIOSIS-Web
URL: http://edina.ac.uk/
Geographical Coverage: Multi-National
Dates Last Searched: 2002; 2006 [Inactive]
Time Coverage: 1996 – 2006
Search Strategy:
#1 SCHIZO*
#2 PSYCHOSIS*
#3 PSYCHOTIC*
#4 PSYCHOSES
#5 OLIGOPHRENI*
#6 HEBEPHRENI*
#7 #1 or #2 or #3 or #4 or #5 or #6

EMBASE
Host: Ovid SP
URL: http://ovidsp.ovid.com
Geographical Coverage: Multi-National
Dates Last Searched: October 21, 2015 [Regular Monthly Updates]
Time Coverage: 1974 – Present
Search Strategy:
1. (clin$ adj2 trial).mp.
2. ((singl$ or doubl$ or trebl$ or tripl$) adj (blind$ or mask$)).mp.
3. (random$ adj5 (assign$ or allocat$)).mp.
4. randomi$.mp.
5. crossover.mp.
6. exp randomized-controlled-trial/
7. exp double-blind-procedure/
8. exp crossover-procedure/
9. exp single-blind-procedure/
10. exp randomization/
11. 1 or 2 or 3 or 4 or 5 or 6 or 7 or 8 or 9 or 10
12. (schizo$ or psychotic$ or psychosis or psychoses).mp.
13. ((chronic$ or severe$ or persistent$) adj (mental$ or psychological$) adj (disorder$ or ill$)).mp.
14. exp schizophrenia/
15. exp psychosis/
16. mental patient/
17. (tardiv$ adj dyskine$).mp.
18. neuroleptic agent/
19. (neuroleptic$ and (malignant adj2 syndrome)).mp.
20. tardive dyskinesia/
21. akathisia/

22. exp neuroleptic malignant syndrome/
23. (neuroleptic$ and movement and disorder$).mp.
24. parkinsoni$.mp.
25. parkinson's.mp.
26. 12 or 13 or 14 or 15 or 16 or 17 or 18 or 19 or 20 or 21 or 22 or 23 or 24 or 25
27. 26 not parkinson's.ti.
28. 11 and 27

Health Research Information Centre
Geographical Coverage: Thailand
Dates Last Searched: November 2008; October 22, 2015 [Inactive]
Time Coverage: Unclear
Search Strategy:
Links to another database, where only the Department of Mental Health Research database can be accessed.

Health Sciences E-index & Clipping: Health Sciences e-Index & Clipping. Naraesuan University
URL: http://mis.lib.nu.ac.th/med_index/
Geographical Coverage: Thailand
Dates Last Searched: November 2008 [We need volunteers to update this search]
Time Coverage: Unclear – Present
Search Strategy:
#1 randomized
#2 randomised
#3 double-blind
#4 สุ่ม
#5 เปรียบเทียบ
#6 #1 or #2 or #3 or #4 or #5

Health Sciences E-Research, Naraesuan University
URL: http://mis.lib.nu.ac.th/med_research/
Geographical Coverage: Thailand
Dates Last Searched: November 2008 [We need volunteers to update this search]
Time Coverage: 1998
Search Strategy:
#1 randomized
#2 randomised
#3 double-blind
#4 สุ่ม
#5 #1 or #2 or #3 or #4

Health System Research Institute Library: **ห้องสมุดสถานบันวิจัยระบบสาธารณะสุข**
URL: http://library.hsri.or.th/en/database.php?
Geographical Coverage: Thailand
Dates Last Searched: November 2008 [We need volunteers to update this search]
Time Coverage: Unclear
Search Strategy:
#1 randomi$
#2 double blind
#3 double-blind
#4 สุ่ม
#5 #1 or #2 or #3 or #4

HEED: Health Economic Evaluations Database

Host: Wiley
URL: http://onlinelibrary.wiley.com/book/10.1002/9780470510933
Geographical Coverage: Multi-National
Dates Last Searched: 2014; October 22, 2015 [Inactive]
Time Coverage: Unclear - 2014
Search Strategy:
#1 schizo*
#2 random*
#3 #1 AND #2

Health Research and Development Information Network (HERDIN)
URL: http://www.herdin.ph/
Geographical Coverage: Philippines
Dates Last Searched: 1995 – Present [Planned Annual Update]
Time Coverage: Unclear – Present
Search Strategy:
#1 random$

HERDIN biomedical bibliographic database
URL: http://www.herdin.ph/old/
Geographical Coverage: Philippines
Dates Last Searched: August 2006; October 22, 2015 [Inactive]
Time Coverage: 1966 – 2006
Search Strategy:
#1 random$

iKnowledge Digital Contents Management Centre SWU
URL: http://dclib.swu.ac.th/main.nsp?view=DCMS
Geographical Coverage: Thailand
Dates Last Searched: November 2008; October 22, 2015 [Inactive]
Time Coverage: Unclear
Search Strategy:
#1 randomi*
#2 double blin*
#3 random*
#4 alloc*
#5 #3 and #4
#6 assign*
#7 #3 and #6
#8 สุ่ม
#9 #1 or #2 or #5 or #7 or #8

iKnowledge Digital Contents Management Centre: Knowledge Digital Contents Management Centre Khon Kaen University
URL: http://dcmskku.kku.ac.th/dcms/main.nsp?view=DCMS
Geographical Coverage: Thailand
Dates Last Searched: December 2008; October 22, 2015 [Inactive]
Time Coverage: Unclear
Search Strategy:
#1 randomi*
#2 double blin*
#3 random*
#4 alloc*
#5 #3 and #4

#6 assign*
#7 #3 and #6
#8 สุ่ม
#9 #1 or #2 or #5 or #7 or #8

IMSEAR: Index Medicus for South-East Asia Region
URL: http://library.searo.who.int/modules.php?op=modload&name=websis&file=imsear
Geographical Coverage: South-East Asia
Dates Last Searched: 2006 [Planned Annual Update]
Time Coverage: Unclear – Present
Search Strategy:
Unclear

IndMED
URL: http://indmed.nic.in/
Geographical Coverage: India
Dates Last Searched: 2004 [Planned Annual Update]
Time Coverage: 1985 – Present
Search Strategy:
#1 randomi$
#2 random$
#3 alloc$
#4 assign$
#5 #3 or #4
#6 #2 and #5
#7double
#8 blind$
#9 mask$
#10 #8 or #9
#11 #7 and #10
#12 crossover
#13 placebo$
#14 #1 or #6 or #11 or #12 or #13

International Pharmaceutical Abstracts
Host: Ovid SP
URL: http://ovidsp.ovid.com/
Geographical Coverage: USA
Dates Last Searched: May 2010 [Planned Annual Update]
Time Coverage: 1970 – Present
Search Strategy:
#1 (clinic$ adj2 trial).mp. [mp=title, subject heading word, registry word, abstract, trade name/generic name]
#2 (random$ adj5 control$ adj5 trial$).mp. [mp=title, subject heading word, registry word, abstract, trade name/generic name]
#3 (crossover or cross-over).mp. [mp=title, subject heading word, registry word, abstract, trade name/generic name]
#4 ((singl$ or double$ or trebl$ or tripl$) adj (blind$ or mask$)).mp. [mp=title, subject heading word, registry word, abstract, trade name/generic name]
#5 randomi$.mp. [mp=title, subject heading word, registry word, abstract, trade name/generic name]
#6 (random$ adj5 (assign$ or allocat$ or assort$ or reciev$)).mp. [mp=title, subject heading word, registry word, abstract, trade name/generic name]
#7 1 or 2 or 3 or 4 or 5 or 6
#8 schizo$.mp. [mp=title, subject heading word, registry word, abstract, trade name/generic name]

#9 hebephreni$.mp. [mp=title, subject heading word, registry word, abstract, trade name/generic name]
#10 oligophreni$.mp. [mp=title, subject heading word, registry word, abstract, trade name/generic name]
#11 psychotic$.mp. [mp=title, subject heading word, registry word, abstract, trade name/generic name]
#12 psychosis.mp. [mp=title, subject heading word, registry word, abstract, trade name/generic name]
#13 psychoses.mp. [mp=title, subject heading word, registry word, abstract, trade name/generic name]
#14 ((chronic$ or sever$) adj2 mental$ adj2 (ill$ or disorder$)).mp. [mp=title, subject heading word, registry word, abstract, trade name/generic name]
#15 (tardiv$ adj dyskine$).mp. [mp=title, subject heading word, registry word, abstract, trade name/generic name]
#16 akathisi$.mp. [mp=title, subject heading word, registry word, abstract, trade name/generic name]
#17 acathisi$.mp. [mp=title, subject heading word, registry word, abstract, trade name/generic name]
#18 (neuroleptic$ and (malignant adj2 syndrome)).mp. [mp=title, subject heading word, registry word, abstract, trade name/generic name]
#19 (neuroleptic$ and (movement and disorder$)).mp. [mp=title, subject heading word, registry word, abstract, trade name/generic name]
#20 parkinsoni$.mp. [mp=title, subject heading word, registry word, abstract, trade name/generic name]
#21 neuroleptic-induc$.mp. [mp=title, subject heading word, registry word, abstract, trade name/generic name]
#22 15 or 16 or 17 or 18 or 19 or 20 or 21
#23 22 not (parkinson's adj1 disease).ti.
#24 8 or 9 or 10 or 11 or 12 or 13 or 14
#25 23 or 24
#26 7 and 25

IranMedex [Now Barakat Knowledge Network System]
URL: http://www.barakatkns.com/
Geographical Coverage: Iran
Dates Last Searched: May 2015 [Planned Annual Update]
Time Coverage: 1966 – Present
Search Strategy:
#1 randomised
#2 randomized
#3 "double blind"
#4 allocated
#5 randomly
#6 #1 or #2 or #3 or #4 or #5
#7 schizophrenia
#8 schizophrenics
#9 psychosis
#10 #7 or #8 or #9
#11 #6 and #10

IRAN-ParsMed
URL: http://www.parsmed.com/
Geographical Coverage: Iran
Dates Last Searched: 2005; October 22, 2015 [Inactive]
Time Coverage: 1995
Search Strategy:
Schizophrenia

J-EAST
URL: http://sciencelinks.jp/j-east/
Geographical Coverage: Japan

Dates Last Searched: 2004; October 22, 2015 [Inactive] [We need volunteers to update this search]
Time Coverage: Unclear – Present
Search Strategy:
#1 schizophrenia/KW
#2schizophrenia/TI
#3 #1 or #2
#4 blind/KW
#5 #3 and #4

Khon Kaen University Library Catalogue
URL: http://kkulib.kku.ac.th/
Geographical Coverage: Thailand
Dates Last Searched: November 2008 [We need volunteers to update this search]
Time Coverage: 1999 – Present
Search Strategy:
Two searches:
Search option: Books, periodicals, etc -
#1 randomi**
#2 double
#3 blind*
#4 #2 and #3
#5 random*
#6 allo*
#7 assign*
#8 #6 or #7
#9 #5 and #8
#10 สุ่ม
#11 เปรียบเทียบ
#12 #1 or #4 or #9 or #10 or #11
#13 #12 in search field medical
Search option: Thai journal index database:
#1 randomi**
#2 double
#3 blind*
#4 #2 and #3
#5 random*
#6 allo*
#7 assign*
#8 #6 or #7
#9 #5 and #8
#10 สุ่ม
#11 #1 or #4 or #9 or #10
#12 #11 in all language, all materials, all regions

KoreaMED
URL: http://www.koreamed.org/
Geographical Coverage: Korea
Dates Last Searched: July 2013
Time Coverage: 1990 – Present
Search Strategy:
#1 randomi*
#2 double-blind*
#3 "double blind"
#4 #1 or #2 or #3

LILACS
URL: http://bases.bireme.br/cgi-bin/wxislind.exe/iah/online/?IsisScript=iah/iah.xis&base=LILACS&lang=i
Geographical Coverage: Latin America and Caribbean Region
Dates Last Searched: 1996
Time Coverage: 1982 – Present
Search Strategy:
#1 RANDOM$
#2 ALEATORI$ or CASUAL or ACASO or AZAR
#3 ((DUPLO or DOBLE or SIMPLE or TRIPLO or TRIPLE) and (CEGO or CIEGO))
#4 ((DOUBL$ or SINGL$ or TRIPL$ or TREBL$) and (BLIND$ or MASK$)
#5 SINGLE-MASKED STUDY/
#6 DOUBLE-MASKED STUDY/
#7 PROPHYLATIC CONTROLLED TRIALS/
#8 PLACEBO$ and CONTROL$
#9 CLINICAL$ and TRIAL$
#10 #1 or #2 or #3 or #4 or #5 or #6 or #7 or #8 or #9

LILACS 2010-ongoing
Host: BIREME
URL: http://bases.bireme.br/cgi-bin/wxislind.exe/iah/online/?IsisScript=iah/iah.xis&base=LILACS&lang=i
Geographical Coverage: Latin America and Caribbean Region
Dates Last Searched: March 23, 2010 [Planned Annual Update]
Time Coverage: 1982 - Present
Search Strategy:
#1 ((Pt RANDOMIZED CONTROLLED TRIAL OR Pt CONTROLLED CLINICAL TRIAL OR Mh RANDOMIZED CONTROLLED TRIALS OR Mh RANDOM ALLOCATION OR Mh DOUBLE-BLIND METHOD OR Mh SINGLE-BLIND METHOD OR Pt MULTICENTER STUDY) OR ((tw ensaio or tw ensayo or tw trial) and (tw azar or tw acaso or tw placebo or tw control$ or tw aleat$ or tw random$ or (tw duplo and tw cego) or (tw doble and tw ciego) or (tw double and tw blind)) and tw clinic$)) AND NOT ((CT ANIMALS OR MH ANIMALS OR CT RABBITS OR CT MICE OR MH RATS OR MH PRIMATES OR MH DOGS OR MH RABBITS OR MH SWINE) AND NOT (CT HUMAN AND CT ANIMALS)) [Words] and schizo$ [Words]
#2 ((Pt ENSAYO CONTROLADO ALEATORIO OR Pt ENSAYO CLINICO CONTROLADO OR Mh ENSAYOS CONTROLADOS ALEATORIOS OR Mh DISTRIBUCIÓN ALEATORIA OR Mh METODO DOBLE CIEGO OR Mh METODO SIMPLECIEGO OR Pt ESTUDIO MULTICÉNTRICO) or ((tw ensaio or tw ensayo or tw trial) and (tw azar or tw acaso or tw placebo or tw control$ or tw aleat$ or tw random$ or (tw duplo and tw cego) or (tw doble and tw ciego) or (tw double and tw blind)) and tw clinic$)) AND NOT ((Ct ANIMALES OR Mh ANIMALES OR Ct CONEJOS OR Ct RATÓN OR MH Ratas OR MH Primates OR MH Perros OR MH Conejos OR MH Porcinos) AND NOT (Ct HUMANO AND Ct ANIMALES)) [Palabras] and Esquizofrenia or Schizo$ or F03.700.750 [Palabras]
3((Pt ENSAIO CONTROLADO ALEATORIO OR Pt ENSAIO CLINICO CONTROLADO OR Mh ENSAIOS CONTROLADOS ALEATORIOS OR Mh DISTRIBUICAO ALEATORIA OR Mh MÉTODO DUPLO-CEGO OR Mh MÉTODO SIMPLES-CEGO or PT ESTUDO MULTICENTRICO) or ((tw ensaio or tw ensayo or tw trial) and (tw azar or tw acaso or tw placebo or tw control$ or tw aleat$ or tw random$ or (tw duplo and tw cego) or (tw doble and tw ciego) or (tw double and tw blind)) and tw clinic$)) AND NOT ((Ct ANIMAIS OR ct coelhos or ct camundongos or MH ANIMAIS OR MH RATOS OR MH PRIMATAS OR MH CAES OR MH COELHOS OR MH SUINOS) AND NOT (Ct HUMANO AND Ct ANIMAIS)) [Palavras] and Esquizofrenia or Schizo$ or F03.700.750 [Palavras]
#4 #1 AND #2 AND #3

Lilly Clinical Trial Registry
URL: http://www.lillytrials.com/
Geographical Coverage: Multi-National
Dates Last Searched: 2006
Time Coverage: Unclear – Present
Note: No longer searching as site redirects clinicaltrials.gov
Search Strategy:
See ClinicalTrials.gov

Lundbeck Trials
URL: http://www.lundbecktrials.com/
Geographical Coverage: Multi-National
Dates Last Searched: October 2010
Time Coverage: Unclear – Present
Search Strategy:
No search facility available

Magyar Orvosi Bibliográfia
URL: http://www.eski.hu/index_en.html
Geographical Coverage: Hungary
Dates Last Searched: 2004 [Planned Annual Update]
Time Coverage: 1989 – Present
Search Strategy:
#1 kontrollált vizsgálat
#2 controlled trial
#3 kontrollált tanulmány
#4 controlled study
#5 randomizált vizsgálat
#6 randomised trial
#7 randomized trial
#8 randomizált kontrollált vizsgálat
#9 randomised (or randomized) controlled trial
#10 randomized controlled trial
#11 randomizált kontrollált tanulmány
#12 randomised controlled study
#13 randomized controlled study
#14 véletlen besorolásos kontrollált vizsgálat
#15 controlled trial with random assignment
#16 véletlen besorolásos vizsgálat
#17 trial with random assignment
#18 véletlen besorolásos tanulmány
#19 study with random assignment
#20 véletlen besorolásos kontrollált tanulmány
#21 controlled study with random assignment
#22 Randomizál
#23 randomizes
#24 #1 or #2 or #3 or #4 or #5 or #6 or #7 or #8 or #9 or #10 or #11 or #12 or #13 or #14 or #15 or #16 or #17 or #18 or #19 or #20 or #21 or #22 or #23

Mahidol University Library and Information Centre: สำนักหอสมุด มหาวิทยาลัยมหิดล
URL: http://www.li.mahidol.ac.th/
Geographical Coverage: Thailand
Dates Last Searched: November 2008 [We need volunteers to update this search]
Time Coverage: Unclear – Present
Search Strategy:

#1 randomi*
#2 double blin*
#3 random*
#4 alloc*
#5 assign*
#6 #4 or #5
#7 #3 and #6
#8 สุ่ม
#9 #1 or #2 or #7 or #8

MEDIC
URL: http://www.terkko.helsinki.fi/medic/
Geographical Coverage: Finland
Dates Last Searched: 2004 [Planned Annual Update]
Time Coverage: Unclear – Present
Search Strategy:
Unclear

MEDLINE
Host: Ovid SP
Geographical Coverage: Multi-National [Focused on English-Speaking World]
URL: http://ovidsp.ovid.com/
Dates Last Searched: October 22, 2015 [Regular Monthly Updates]
Time Coverage: 1946 – Present
Search Strategy:
1. exp clinical trials/
2. exp randomized controlled trials/
3. exp double-blind method/
4. exp single-blind method/
5. exp cross-over studies/
6. randomized controlled trial.pt.
7. clinical trial.pt.
8. controlled clinical trial.pt.
9. (clinic$ adj2 trial).mp.
10. (random$ adj5 control$ adj5 trial$).mp.
11. (crossover or cross-over).mp.
12. ((singl$ or double$ or trebl$ or tripl$) adj (blind$ or mask$)).mp.
13. randomi$.mp.
14. (random$ adj5 (assign$ or allocat$ or assort$ or reciev$)).mp.
15. or/1-14
16. exp SCHIZOPHRENIA/
17. exp Paranoid Disorders/
18. schizo$.mp.
19. hebephreni$.mp.
20. oligophreni$.mp.
21. psychotic$.mp.
22. psychosis.mp.
23. psychoses.mp.
24. ((chronic$ or sever$) adj2 mental$ adj2 (ill$ or disorder$)).mp.
25. exp dyskinesia, drug-induced/
26. exp psychomotor agitation/
27. exp neuroleptic malignant syndrome/
28. exp "diagnosis, dual (psychiatry)"/
29. (tardiv$ adj dyskine$).mp.

30. akathisi$.mp.
31. acathisi$.mp.
32. (neuroleptic$ and (malignant adj2 syndrome)).mp.
33. (neuroleptic$ and (movement and disorder$)).mp.
34. parkinsoni$.mp.
35. neuroleptic-induc$.mp.
36. or/29-35
37. 36 not (parkinson's adj1 disease).ti.
38. or/16-28
39. 37 or 38
40. 15 and 39

Mental Health & Psychiatric Nursing Abstract: **ฐานข้อมูลวิจัยทางสุขภาพจิตและจิตเวช**
URL: http://www.dmh.moph.go.th/abstract/nurse.asp
Geographical Coverage: Thailand
Dates Last Searched: November 2008 [We need volunteers to update this search]
Time Coverage: Unclear – Present
Search Strategy:
#1 randomi
#2 double-blind
#3 สุ่ม
#4 เปรียบเทียบ
#5 #1 or #2 or #3 or #4

Mental Health and Psychiatric Abstract Database
URL: http://www.dmh.moph.go.th/abstract/
Geographical Coverage: Thailand
Dates Last Searched: November 2008 [We need volunteers to update this search]
Time Coverage: Unclear – Present
Search Strategy:
#1 randomi
#2 random
#3 double-blind
#4 สุ่ม
#5 #1 or #2 or #3 or #4

Midwifery and Infant Care: Maternity and Infant Care
URL: http://arc.uk.ovid.com/
Dates Last Searched: May 2006; October 22, 2015 [Inactive]
Time Coverage: Unclear
Search Strategy:
#1 random*
#2 clinical trial*
#3 double?blind*
#4 trial*
#5 #1 or #2 or #3 or #4
#6 dyskines*
#7 a?athisia
#8 neuroleptic malignant syndrom*
#9 schizo*
#10 psychotic*
#11 psychos?s
#12 #6 or #7 or #8 or #9 or #10 or #11
#13 #5 and #12

National Alliance for Research: National Alliance for Research on Schizophrenia and Depression
URL: http://www.mhsource.com/narsad/bd/studyops.html
Geographical Coverage: USA
Dates Last Searched: February 2001; October 22, 2015 [Inactive]
Time Coverage: Unclear
Search Strategy:
#1 schiz*

National Institutes of Health
URL: http://www.nih.gov/
Geographical Coverage: USA
Dates Last Searched: 2006
Time Coverage: Unclear
Search Strategy:
#1 schiz*
#2 randomi*
#3 #1 and #2

National Research Council of Thailand Library: **ห้องสมุดงานวิจัย สำนักงานคณะกรรมการวิจัยแห่งชาติ**
URL: http://www.riclib.nrct.go.th/index_e.html
Geographical Coverage: Thailand
Dates Last Searched: November 2008 [We need volunteers to update this search]
Time Coverage: Unclear – Present
Search Strategy:
#1 randomi$
#2 double-blind
#3 สุ่ม
#4 เปรียบเทียบ
#5 #1 or #2 or #3 or #4

National Research Register
Geographical Coverage: UK
Dates Last Searched: March 2001; October 22, 2015 [Inactive]
Time Coverage: Unclear
Note: The UK National Research Register is an archived site and is no longer being updated.
Search Strategy:
#1 randomi*
#2 double*
#3 #1 or #2
#4 schiz*
#5 psychoses
#6 psychosis
#7 psychotic*
#8 #4 or #5 or #6 or #7
#9 #3 and #8

National Technical Information Service
URL: http://www.ntis.gov/search/index.aspx
Geographical Coverage: USA
Dates Last Searched: May 2010 [Planned Annual Update]
Time Coverage: Unclear – Present
Search Strategy:

Search option: title
#1 Schizophrenia

Office of Research Development, PCM & PKH: สำนักงานพัฒนางานวิจัย วิทยาลัยแพทยศาสตร์พระมงกุฎเกล้า/โรงพยาบาลพระมงกุฎเกล้า
URL: http://research.pcm.ac.th/research/datatitle.php
Geographical Coverage: Thailand
Dates Last Searched: November 2008; October 22, 2015 [Inactive]
Time Coverage: Unclear
Search Strategy:
#1 randomized
#2 double blind
#3 #1 or #2
#4 #3 in English title field
#5 randomized
#6 double blind
#7 a Thai term
#8 #5 or #6 or #7
#9 #8 in Thai title field
#10 #4 and #9

Panteleimon: Medical and biological databases
URL: http://www.panteleimon.org/
Geographical Coverage: Ukraine
Dates Last Searched: August 25, 2004 [Planned Annual Update]
Time Coverage: Unclear – Present
Search Strategy:
Search option: Abstract, title, article, keyword:
#1 randomi
#2 double-blind
#3 allocate
#4 assign
#5 clinical trial
#6 placeo
#7 crossover
#8 Рандомиз
#9 двойного слепого
#10 распределить
#11 определить
#12 клиническое исследование
#13 плацебо
#14 скрестить
#15 перекрест
#16 Рандомиз
#17 ДВОЇСТИЙ СЛІПИЙ
#18 РОЗПОДІЛИТИ
#19 ВИЗНАЧИТИ
#20 КЛІНІЧНА ДОСЛІДЖЕННЯ
#21 плацебо
#22 СХРЕСТИТИ
#23 ПЕРЕХРЕСТ
#24 #1 or #2 or #3 or #4 or #5 or #6 or #7 or #8 or #9 or #10 or #11 or #12 or #13 or #14 or #15 or #16 or #17 or #18 or #19 or #20 or #21 or #22 or #23

POPLINE
Host: K4Health project
URL: http://www.popline.org/
Geographical Coverage: USA
Dates Last Searched: November 01, 2010 [Planned Annual Update]
Time Coverage: 1973 – Present
Search Strategy:
Schizo* / psychosis / psychoses / psychotic / tardive dyskinesia /akathisia

Prince of Songkla E-Research Database
URL: http://kb.psu.ac.th/psukb/
Geographical Coverage: Thailand
Dates Last Searched: November 2008 [Planned Annual Update]
Time Coverage: Unclear
Search Strategy:
#1 randomised
#2 randomized
#3 double blind
#4 สุ่ม
#5 #1 or #2 or #3 or #4

PsycEXTRA
URL: http://psycnet.apa.org/index.cfm?fa=search.advancedSearchForm
Geographical Coverage: Multi-National
Dates Last Searched: February 25, 2008 [Planned Annual Update]
Time Coverage: 1908 – Present
Search Strategy:
1 exp clinical trials/ or experimental design/ (443)
2 exp treatment effectiveness evaluation/ (404)
3 exp mental health program evaluation/ (38)
4 exp random sampling/ (2)
5 randomi$.mp. (450)
6 (clinic$ adj4 trial$).mp. (764)
7 (random$ adj5 (assign$ or allocat$ or assort$)).mp. (266)
8 (crossover or cross-over).mp. (31)
9 ((singl$ or doubl$ or tripl$ or trebl$) adj (blind$ or mask$)).mp. (86)
10 exp placebo/ (43)
11 placebo$.mp. (273)
12 1 or 2 or 3 or 4 or 5 or 6 or 7 or 8 or 9 or 10 or 11 (1993)
13 exp psychosis/ (567)
14 exp schizophrenia/ (469)
15 exp schizoaffective disorder/ (21)
16 exp dyskinesia/ (6)
17 exp movement disorders/ (116)
18 exp akathisia/ (0)
19 exp neuroleptic malignant syndrome/ (0)
20 exp parkinsonism/ (2)
21 schizo$.mp. (694)
22 hebephreni$.mp. (1)
23 oligophreni$.mp. (0)
24 psychotic$.mp. (168)
25 psychos#s$.mp. (209)
26 ((chronic$ or sever$) adj2 mental$ adj2 (ill$ or disorder$)).mp. (236)
27 (tardiv$ adj dyskine$).mp. (11)

28 (akathisi$ or acathisi$).mp. (1)
29 (neuroleptic$ adj4 malignan$ adj4 syndrom$).mp. (2)
30 (movement adj5 disorder$).mp. (27)
31 parkinsoni$.mp. (12)
32 neuroleptic-induc$.mp. (1)
33 (parkinson?s adj disease).ti. (48)
34 13 or 14 or 15 or 21 or 22 or 23 or 24 or 25 or 26 (1131)
35 16 or 17 or 18 or 19 or 20 or 27 or 28 or 29 or 30 or 31 or 32 (141)
36 35 not 33 (139)
37 34 or 36 (1258)
38 12 and 37 (45)
39 from 38 keep 1-45 (45)

Psychiatry
URL: http://www.psikiyatridizini.org/
Geographical Coverage: Turkey
Dates Last Searched: June 2006 [Planned Annual Update]
Time Coverage: 1968
Search Strategy:
#1 Randomi
#2 allocated
#3 double blind
#4 double-blind
#5 #1 or #2 or #3 or #4

PsycINFO
Host: Ovid SP
URL: http://www.apa.org/psycinfo/
Geographical Coverage: Multi-National
Dates Last Searched: October 22, 2015 [Regular Monthly Updates]
Time Coverage: 1806 – Present
Search Strategy:
1. randomi$.mp.
2. ((singl$ or doubl$ or trebl$ or tripl$) adj (blind$ or mask$)).mp.
3. placebo$.mp.
4. exp placebo/
5. crossover.mp.
6. exp treatment effectiveness evaluation/
7. exp mental health program evaluation/
8. (random$ adj (assign$ or allocate$)).mp.
9. or/1-8
10. schizo$.mp.
11. hebephreni$.mp.
12. oligophreni$.mp.
13. psychotic$.mp.
14. psychosis.mp.
15. psychoses.mp.
16. ((chronic$ or sever$) adj2 mental$ adj2 (ill$ or disorder$)).mp.
17. exp psychosis/
18. exp schizophrenia/
19. exp schizoaffective disorder/
20. (tardiv$ adj dyskine$).mp.
21. akathisi$.mp.
22. acathisi$.mp.
23. (neuroleptic$ and (malignant adj2 syndrome)).mp.

24. (neuroleptic$ and (movement and disorder$)).mp.
25. exp neuroleptic malignant syndrome/
26. exp dyskinesia/
27. exp tardive dyskinesia/
28. exp akathisia/
29. neuroleptic-induc$.mp.
30. parkinsoni$.mp.
31. parkinsonism-.sh.
32. (parkinson's adj1 disease).ti.
33. or/10-31
34. 33 not 32
35. 9 and 34

PsycLIT
Geographical Coverage: USA
Dates Last Searched: 1996; October 22, 2015 [Inactive]
Time Coverage: 1974
Note: It was merged into the PsycINFO online database in 2000.
Search Strategy:
Trial search phrase:
#1 randomi*
#2 singl*
#3 doubl*
#4 trebl*
#5 tripl*
#6 blind*
#7 mask*
#8 (#2 or #3 or #4 or #5) near (#6 or #7)
#9 clin*
#10 trial*
#11 #9 near #10
#12 placebo*
#13 placebo- in de
#14 crossover
#15 treatment-effectiveness-evaluation in de
#16 mental-health-program-evaluation in de
#17 random*
#18 assign*
#19 allocate*
#20 #17 near (#18 or #19)
#21 #20 or #16 or #15 or #14 or #13 or #12 or #11 or #8 or #1
schizophrenia search phrase:
#1 schizo*
#2 hebephreni*
#3 oligophreni*
#4 psychotic*
#5 psychosis
#6 psychoses
#7 chronic*
#8 sever*
#9 mental*
#10 ill*
#11 disorder*
#12 ((chronic* or sever*) near2 mental*) near2 (ill* or disorder*)
#13 explode "schizophrenia"

#14 explode "psychosis"
#15 explode "schizoaffective-disorder"
#16 #1 or #2 or #3 or #4 or #5 or #6 or #12 or #13 or #14 or #15
#17 tardiv*
#18 dyskine*
#19 tardiv* near dyskine*
#20 akathisi*
#21 acathisi*
#22 neuroleptic*
#23 malignant
#24 syndrome
#25 neuroleptic* and (malignant near2 syndrome)
#26 movement
#27 disorder*
#28 #22 and movement and disorder*
#29 parkinsoni*
#30 neuroleptic-induc*
#31 parkinson's
#32 disease
#33 parkinson's near1 (disease in ti)
#34 #19 or #20 or #21 or #25 or #28 or #29 or #30
#35 #34 not #33
#36 "neuroleptic-malignant-syndrome" in de
#37 explode "dyskinesia"
#38 explode "akathisia"
#39 "parkinsonism-" in de
#40 #35 or #36 or #37 or #38 or #39
#41 #40 or #16
(Journal Articles 1/1974 - 12/1996; Chapters and Books 1/1987 - 12/1996)

PSYINDEX
Geographical Coverage: Germany, Switzerland, Austria
Dates Last Searched: September 2009 [Planned Annual Update]
Time Coverage: 1977 – Unclear
Search Strategy:
#1 random* and (allocat* or assign* or zugewiesen or zugeteilt or eingeteilt) and (UD=199501-200204)
#2 (randomi* or zufall* or zufaell*) and (UD=199501-200204)
#3 ((doubl* or doppel* or singl* or einfach* or tripl* or trebl* or dreifach*) near (blind* or mask*)) and (UD=199501-200204)
#4 pla?ebo* and (treat* or behand* or untersuch*) and (UD=199501-200204)
#5 (doppelblind* or verblind*) and (UD=199501-200204)
#6 (pla?ebo* near ((vs or versus or gegen) or verum)) and (UD=199501-200204) and (UD=199501-200204)
#7 (zufa?ll* or random*) and (experiment* or evalu* or effe?t* or wirk*) and (behand* or untersuch* or treat*) and (UD=199501-200204)
#8 (allocat* or assign* or zugewiesen or zugeteilt or eingeteilt) and (control group* or kontrollgruppe* or beobachtungsgruppe*) and (UD=199501-200204)
#9 random* and (controll* or kontroll*) and (studie* or trial*) and (UD=199501-200204)
#10 #1 or #2 or #3 or #4 or #5 or #6 or #7 or #8 or #9
#11 random* and (controll* or kontroll*) and (UD=199501-200204) and (UD=199501-200204)
#12 #1 or #2 or #3 or #4 or #5 or #6 or #7 or #8 or #11

PubMed
URL: http://www.ncbi.nlm.nih.gov/pubmed

Geographical Coverage: Multi-National [Focused on English-Speaking World]
Dates Last Searched: October 22, 2015 [Regular Monthly Updates]
Time Coverage: 1946 – Present
Search Strategy:
1# schizophrenia[MeSH Terms]
2# paranoid disorders[MeSH Terms]
3# dyskinesia, drug -induced[MeSH Terms]
4# psychomotor agitation[MeSH Terms]
5# diagnosis, dual psychiatry[MeSH Terms]
6# psychotic disorders[MeSH Terms]
7# akathisia drug induced[All Fields]
8# neuroleptic malignant syndrome[MeSH Terms]
9# #1 OR #2 OR #3 OR #4 OR #8 OR #9 OR #10 OR #11
10# hebephreni*
11# oligophreni*
12# psychotic*
13# psychosis
14# psychoses
15# akathisi*
16# acathisi*
17# parkinsoni*
18# neuroleptic-induc*
19# #10 OR #11 OR #12 OR #13 OR #14 OR #15 OR #16 OR #17 OR #18
20# tardiv*
21# dyskine*
22# #23 AND #24
23# neuroleptic*
24# malignant
25# syndrome
26# #23 AND #24
27# #26 and #25
28# movement*
29# #23 and #28
30# disorder*
31# #29 and #30
32# #9 OR #19 OR #22 OR #27 OR #31
33# parkinson's disease [ti]
34# parkinson disease [ti]
35# #35 OR #34
36# #32 NOT #35
37# randomized controlled trial [pt]
38# controlled clinical trial [pt]
39# randomized [tiab]
40# placebo [tiab]
41# drug therapy [sh]
42# randomly [tiab]
43# trial [tiab]
44# groups [tiab]
45# #37 OR #38 OR #39 OR #40 OR #41 OR #42 OR #43 OR #44
46# humans [mh]
47# #45 AND #46
48# #36 AND #47

Russian Medical Journal Search
URL: http://www.rmj.ru/main.htm

Geographical Coverage: Russia
Dates Last Searched: October 01, 2004 [We need volunteers to update this search]
Time Coverage: 1995 – Present
Search Strategy:
#1 Рандомиз
#2 двойно слепо
#3 плацебо
#4 клиническое исследование
#5 определить
#6 распределить
#7 Распредел*
#8 скрестить
#9 Перекрест*
#10 #1 or #2 or #3 or #4 or #5 or #6 or #7 or #8 or #9

RUSSMED
URL: http://www.russmed.ru/eng/ramn.htm
Geographical Coverage: Russia
Dates Last Searched: 1999 [We need volunteers to update this search]
Time Coverage: 1960 – Present
Search Strategy:
Unclear

SIGLE [Now OpenSIGLE and OpenGrey]
URL: http://www.opengrey.eu/
Geographical Coverage: Europe
Dates Last Searched: December 2004 [Planned Annual Update]
Time Coverage: 1980 – Present
Search Strategy:
Trial search phrase:
#1 clin*
#2 trial*
#3 #1 near #2
#4 singl*
#5 doubl*
#6 trebl*
#7 tripl*
#8 blind*
#9 mask*
#10 (#4 or #5 or #6 or #7) near (#8 or #9)
#11 randomi*
#12 random*
#13 allocat*
#14 assign*
#15 #12 near (#13 or #14)
#16 crossover
#17 #16 or #15 or #11 or #10 or #3
schizophrenia search phrase:
#1 schizo*
#2 hebephreni*
#3 oligophreni*
#4 psychotic*
#5 psychosis
#6 psychoses
#7 chronic*

#8 sever*
#9 mental*
#10 ill*
#11 disorder*
#12 ((chronic* or sever*) near2 mental*) near2 (ill* or disorder*)
#13 #1 or #2 or #3 or #4 or #5 or #6 or #12
#14 tardiv*
#15 dyskine*
#16 tardiv* near dyskine*
#17 akathisi*
#18 acathisi*
#19 neuroleptic*
#20 malignant
#21 syndrome
#22 neuroleptic* and (malignant near2 syndrome)
#23 movement
#24 disorder*
#25 #19 and movement and disorder*
#26 parkinsoni*
#27 neuroleptic-induc*
#28 parkinson's
#29 disease
#30 parkinson's near1 (disease in ti)
#31 #16 or #17 or #18 or #22 or #25 or #26 or #27
#32 #31 not #30
#33 #32 or #13

SocioFile [Now Sociological Abstracts]
Host: ProQuest
URL: http://search.proquest.com/
Dates Last Searched: September 11, 2007 [Planned Annual Update]
Time Coverage: 1973 – Present
Search Strategy:
Trial search phrase:
#1 randomi*
#2 clin*
#3 trial*
#4 clin* near trial*
#5 singl*
#6 doubl*
#7 tripl*
#8 trebl*
#9 mask*
#10 blind*
#11 (singl* or doubl* or tripl* or trebl*) and (mask* or blind*)
#12 crossover
#13 random*
#14 allocate*
#15 assign*
#16 random* near (allocate* or assign*)
#17 #16 or #12 or #11 or #4 or #1
schizophrenia search phrase:
#1 explode "schizophrenia"
#2 explode "psychosis"
#3 schizo*

#4 psychosis
#5 psychotic*
#6 psychoses
#7 chronic*
#8 sever*
#9 mental*
#10 ill*
#11 disorder*
#12 (chronic* or sever*) and (mental* and (ill* or disorder*))
#13 oligophreni*
#14 hebephreni*
#15 #1 or #2 or #3 or #4 or #5 or #6 or #12 or #13 or #14
#16 tardiv*
#17 dyskine*
#18 tardiv* near dyskine*
#19 akathisi*
#20 acathisi*
#21 neuroleptic*
#22 malignant
#23 syndrome
#24 neuroleptic* and (malignant near2 syndrome)
#25 movement
#26 disorder*
#27 #21 and movement and disorder*
#28 parkinsoni*
#29 neuroleptic-induc*
#30 parkinson's
#31 disease
#32 parkinson's near1 (disease in ti)
#33 #18 or #19 or #20 or #24 or #27 or #28 or #29
#34 #33 not #32
#35 #34 or #15

Stanley Database
URL: http://www.stanleyresearch.org/
Geographical Coverage: USA
Dates Last Searched: February 2011 [Planned Annual Update]
Time Coverage: 1999 – Present
Search Strategy:
#1 Schizophrenia
#2 psychosis
#3 #1 and #2

STEB: Scientific and Technical Egyptian Bibliographic Database
URL: http://www.sti.sci.eg/enstinetdatabases.htm
Geographical Coverage: Egypt
Dates Last Searched: 2000; October 22, 2015 [Inactive]
Time Coverage: Unclear
Search Strategy:
#1 randomi$
#2 double-blind
#3 assign$
#4 allocate$
#5 #1 or #2 or #3 or #4

Thai Index Medicus: ข้อมูลวารสารทางการแพทย์
URL: http://cuml.md.chula.ac.th/index.shtml
Geographical Coverage: Thailand
Dates Last Searched: November 2008 [Planned Annual Update]
Time Coverage: 1991 – Present
Search Strategy:
Search option: All fields
#1 randomi$
#2 double
#3 blind$
#4 #2 and #3
#5 random$
#6 alloc$
#7 assign$
#8 สุ่ม$
#9 เปรียบเทียบ$
#10 #8 or #9
#11 #10 not #1

Thai Journal Citation Index Centre: ศูนย์ดัชนีอ้างอิงวารสารไทย
URL: http://www.kmutt.ac.th/jif/public_html/searchp.html
Geographical Coverage: Thailand
Dates Last Searched: November 2008 [Planned Annual Update]
Time Coverage: 1996 – Present
Search Strategy:
#1 randomi
#2 double blind
#3 #1 or #2

Thai Medical Index: ข้อมูลวารสารทางการแพทย์
URL: http://www2.medlib.si.mahidol.ac.th/elib/cgi-bin/opacexe.exe?op=gsf&frm=simsch&db=Medindex&skin=su
Geographical Coverage: Thailand
Dates Last Searched: March 2007 [Planned Annual Update]
Time Coverage: 1990 – Present
Search Strategy:
Search option: general keyword
#1 randomi*
#2 double blind*
#3 #1 or #2
#4 สุ่ม
#5 #4 NOT #1
#6 #3 or #5

Thai Research: ฐานข้อมูลวิจัยไทย
URL: http://www.thairesearch.in.th/
Geographical Coverage: Thailand
Dates Last Searched: December 2008; October 22, 2015 [Inactive]
Time Coverage: Unclear
Search Strategy:
#1 randomized
#2 randomised
#3 double blind

#4 สุ่ม
#5 #1 or #2 or #3 or #4

Thai Thesis online: วิทยานิพนธ์ไทย
URL: http://thesis.stks.or.th/
Geographical Coverage: Thailand
Dates Last Searched: November 2008 [Planned Annual Update]
Time Coverage: Unclear – Present
Search Strategy:
#1 randomized
#2 double blind
#3 สุ่ม
#4 #1 or #2 or #3

ThaiLis
URL: http://uc.thailis.or.th/main/index.aspx and http://dcms.thailis.or.th/tdc/
Geographical Coverage: Thailand
Dates Last Searched: November 2008
Time Coverage: 1998 – Present
Search Strategy:
Search option: In all universities, all field, in 'some words'
#1 randomi
#2 double blind
#3 #1 or #2

The Cochrane Library
URL: http://www.cochranelibrary.com/
Geographical Coverage: Multi-National
Dates Last Searched: September 2012 [Planned Annual Update]
Time Coverage: 1995 – Present
Search Strategy:
#1 MeSH descriptor Schizophrenia, this term only
#2 MeSH descriptor Paranoid Disorders, this term only
#3 schizo* in Clinical Trials
#4 hebephreni* in Clinical Trials
#5 oligophreni* in Clinical Trials
#6 psychotic* in Clinical Trials
#7 psychosis in Clinical Trials
#8 psychoses in Clinical Trials
#9 chronic* in Clinical Trials
#10 sever* in Clinical Trials
#11 mental* in Clinical Trials
#12 ill* in Clinical Trials
#13 disorder* in Clinical Trials
#14 (#9 OR #10)
#15 (#14 AND #11)
#16 (#12 OR #13)
#17 (#15 AND #16)
#18 (#1 OR #2 OR #3 OR #4 OR #5 OR #6 OR #7 OR #8 OR #17)
#19 tardiv* in Clinical Trials
#20 dyskine* in Clinical Trials
#21 (#19 AND #20)
#22 akathisi* in Clinical Trials
#23 acathisi* in Clinical Trials

#24 neuroleptic* in Clinical Trials
#25 malignant in Clinical Trials
#26 syndrome in Clinical Trials
#27 (#25 AND #26)
#28 (#24 AND #27)
#29 movement in Clinical Trials
#30 disorder* in Clinical Trials
#31 (#24 AND #29 AND #30)
#32 parkinsoni* in Clinical Trials
#33 neuroleptic-induc* in Clinical Trials
#34 parkinson's in Clinical Trials
#35 disease in Clinical Trials
#36 disease:ti in Clinical Trials
#37 (#34 AND #36)
#38 (#21 OR #22 OR #23 OR #28 OR #31 OR #32 OR #33)
#39 (#38 AND NOT #37)
#40 MeSH descriptor Dyskinesia, Drug-Induced, this term only
#41 MeSH descriptor Akathisia, Drug-Induced, this term only
#42 MeSH descriptor Neuroleptic Malignant Syndrome, this term only
#43 (#39 OR #40 OR #41 OR #42)
#44 (#43 OR #18)
#45 MeSH descriptor Schizophrenia and Disorders with Psychotic Features, this term only
#46 (#45 OR #44)

The Thailand Research Fund's Collection: สำนักงานกองทุนสนับสนุนการวิจัย
URL: http://elibrary.trf.or.th/default.asp
Geographical Coverage: Thailand
Dates Last Searched: November 2008 [Planned Annual Update]
Time Coverage: Unclear – Present
Search Strategy:
#1 randomized
#2 randomised
#3 double-blind
#4 สุ่ม
#5 #1 or #2 or #3 or #4

UKCRN Portfolio Database
URL: http://public.ukcrn.org.uk/search/
Geographical Coverage: UK
Dates Last Searched: January 22, 2014 [Now Not Getting Updated]
Time Coverage: Unclear – Present
Search Strategy:
Select Mental Health

Web of Science
Host: Web of Knowledge
URL: http://isiknowledge.com/
Geographical Coverage: Multi-National [Focused on English-Speaking World]
Dates Last Searched: December 01, 2010 [Planned Annual Update]
Time Coverage: 1900 – Present
Search Strategy:
1 Topic=(schizo*) OR Title=(schizo*)
2 Topic=(psychosis*) OR Title=(psychosis*)
3 Topic=(psychotic*) OR Title=(psychotic*)

4 Topic=(psychoses*) OR Title=(psychoses*)
5 Topic=(oligophreni*) OR Title=(oligophreni*)
6 Topic=(hebephreni*) OR Title=(hebephreni*)
7 Topic=(tardiv*) OR Title=(tardiv*)
8 Topic=(dyskine*) OR Title=(dyskine*)
9 #8 AND #7
10 Topic=(akathisi*) OR Title=(akathisi*)
11 Topic=(acathisi*) OR Title=(acathisi*)
12 Topic=(neuroleptic malignant syndrome*) OR Title=(neuroleptic malignant syndrome*)
13 Topic=(severe mental illness*) OR Title=(severe mental illness*)
14 Topic=(chronic mental illness*) OR Title=(chronic mental illness*)
15 Topic=(chronic mental disorder*) OR Title=(chronic mental disorder*)
16 Topic=(severe mental disorder*) OR Title=(severe mental disorder*)
17 Topic=(neuroleptic-induc*) OR Title=(neuroleptic-induc*)
18 Topic=(parkinson's disease) OR Title=(parkinson's disease)
19 #17 OR #16 OR #15 OR #14 OR #13 OR #12 OR #11 OR #10 OR #9 OR #6 OR #5 OR #4 OR #3 OR #2 OR #1
20 #19 NOT #18
21 Topic=(randomi*) OR Title=(randomi*)
22 Topic=(clin*) OR Title=(clin*)
23 Topic=(trial*) OR Title=(trial*)
24 #23 AND #22
25 TS=(singl* OR Doubl* OR Tripl* OR Trebl*) OR TI=(singl* OR Doubl* OR Tripl* OR Trebl*)
26 TS=(mask* OR blind*) OR TI=(mask* OR blind*)
27 TS=crossover* OR TI=crossover*
28 TS=(allocate* OR assign*) OR TI=(allocate* OR assign*)
29 TS=random* OR TI=random*
30 #29 AND #28
31 #30 OR #27 OR #26 OR #25 OR #24 OR #21
32 #31 AND #20

WHO International Clinical Trials Registry Platform (ICTRP)
Host: WHO
URL: http://apps.who.int/trialsearch/AdvSearch.aspx
Geographical Coverage: Multi-National
Dates Last Searched: October 22, 2015 [Regular Monthly Updates]
Time Coverage: 2000 – Present
Note: This sources includes 16 clinical trial registries.
Search Strategy:
Recruitment status is ALL
Schizophrenia OR Schizotypal OR Schizoaffective OR Schizophreniform OR Psychosis OR Psychoses OR Psychotic OR Tardive OR Dyskinesia OR Akathisia

WHO Representative to Thailand Library: Office of the WHO Representative to Thailand Library
URL: Unclear
Geographical Coverage: Thailand
Dates Last Searched: November 2008
Time Coverage: Unclear
Search Strategy:
#1 randomized
#2 randomised
#3 double-blind
#4 สุ่ม

#5 #1 or #2 or #3 or #4

WHO: EMRO Virtual Health Sciences Library
URL: http://www.emro.who.int/entity/information-resources/index.html
Dates Last Searched: 2005 [Planned Annual Update]
Time Coverage: Unclear – Present
Search Strategy:
#1 randomly
#2 randomi$
#3 allocat$
#4 assign$
#5 placebo
#6 double-blind$
#7 double
#8 blind
#9 #7 or #8
#10 #1 or #2 or #3 or #4 or #5 or #6 or #9

Conference Proceedings
Conference proceedings are searched using a combination of hand searching and electronic searches. Where proceedings are supplied in electronic form these are searched using the following search phrase:
#1 RANDOMI*
#2 RANDOMLY
#3 (RANDOM* AND ALLOC*)
#4 CROSSOVER
#5 ((SINGL* or DOUBL* or TRIPL* or TREBL*) and (BLIND* or MASK*))
#6 (CLIN* AND TRIAL*)
#7 META-ANALYSIS
#8 (PLACEBO* AND CONTROL*)]
#9 #1 or #2 or #3 or #4 or #5 or #6 or #7 or #8
The results of electronic searches are then hand searched. All reports likely to relate to a trial relevant to the scope of the Group are obtained and added to the Group's Register. All other trials are forwarded to the Cochrane Central Register of Controlled Trials (formerly known as the Cochrane Controlled Trials Register).

List of Proceedings
Annual Congress of the International Society of Psychoneuroendocrinology
25th Annual Congress; 1994 Aug 14-18; Seattle, Washington, USA
30th Annual Congress; 1999 Jul 30 - Aug 3; Orlando, Florida, USA
Annual Meeting of the College of Psychiatric and Neurologic Pharmacists
2nd Annual Meeting; 1999 Mar 25-28; Lake Tahoe, California, USA
Annual Meeting of the International Society of Technology Assessment in Health Care
12th Annual Meeting; 1996 Jun 23-26; San Francisco, California USA
18th Annual Meeting; 2002 Jun 9-12; Berlin, Germany
Annual Meeting of the New Clinical Drug Evaluation Unit
21st Annual Meeting; 1981 May 26-28; Key Biscane, Florida, USA
Annual Meeting of the Society for Neuroscience
27th Annual Meeting; 1997 Oct 25-30; New Orleans, Louisiana, USA
30th Annual Meeting; 2000 Nov 4-9; New Orleans, Louisiana, USA
31st Annual Meeting; 2001 Nov 10-15; San Diego, California, USA
Annual Meeting of the Society for Research on Nicotine and Tobacco
6th Annual Meeting; 2000 Feb 18-20; Arlington, Virginia, USA
9th Annual Meeting; 2003 Feb 19-22; New Orleans, Louisiana, USA
10th Annual Meeting; 2004 Feb 18-21; Phoenix, Arizona, USA

Collegium Internationale Neuro-Psychopharmacologicum Regional Meeting
1997 Aug 21-23; Acapulco, Mexico
Congres de Neuropsychopharmacologie
9th Congres - Nouveaux Developpements du Sulpiride; 1974 Jul 10; Paris, France
Congres de Psychiatrie et de Neurologie de Langue Francaise
86th Congres; 1988 Jun 13-17; Chambery, France
96th Congres; 1998 May 10-15; Saint Paul, France
International Congress of Endocrinology
9th International Congress; 1992 Aug 30 - Sep 5; Nice, France
International Risperidone Investigators' Meeting
1st International Risperidone Investigators' Meeting; 1992 Mar 9-10; Paris, France
International Symposium for the Psychological Treatment of Schizophrenia and other Psychoses
13th International Symposium; 2000 Jun 5-9; Stavanger, Norway
International Workshop on Brain Uptake and Utilization of Fatty Acids
2000 Mar 2-4; Bethesda, Maryland, USA
Keio University International Symposia for Life Sciences and Medicine on Comprehensive Treatment of Schizophrenia
8th Keio University International Symposia - Linking Neurobehavioural Findings to Psychosocial Approaches; 2000 Jun 5-7; Tokyo, Japan
Symposium der Arbeitsgemeinschaft fuer Neuropsychopharmakologie und Pharmakopsychiatrie
1981; Nuernberg, Germany
Treatment-resistant schizophrenia and beyond: current concepts and future prospects
1998 Jul 8-9; London, UK

Hand Searching
Acta Psychiatrica Scandinavica - Start: 1948 Last year done: 2008 Ongoing: yes
Acta Psychiatrica Scandinavica Supplementum - Start: 1948 Last year done: 2008 Ongoing: yes
American Journal of Psychiatry - Start: 1948 Last year done: 2008 Ongoing: yes
Arab Journal of Psychiatry - Start: 1990 Last year done: 1997 Ongoing: no
Archives of General Psychiatry - Start: 1959 Last year done: 2008 Ongoing: no
Australian and New Zealand Journal of Psychiatry - Start: 1967 Last year done: Ongoing: no
Behaviour Therapy - Start: 1971 Last year done: 1976, 1981, 1986, 1991 Ongoing: no
Biological Psychiatry - Start: 1994 Last year done: 1995 Ongoing: no
British Journal of Psychiatry - Start: 1963 Last year done: 2008 Ongoing: yes
Canadian Journal of Psychiatry - Start: 1979 Last year done: Ongoing: no
Canadian Psychiatric Association Journal (continued as Canadian Journal of Psychiatry - Start: 1956 Last year done: 1978 Ongoing: no
Clinical Pharmacology and Therapeutics - Start: 1971 Last year done: 1976, 1981, 1986, 1991 Ongoing: no
Clinical Schizophrenia and Related Psychoses – Start: 2008 Last year done 2015 Ongoing: yes
Der Nervenarzt - Start: 1980 Last year done: 1995 Ongoing: no
Egyptian Journal of Mental Health - Start: 1988 Last year done: 1990-1992 Ongoing: no
Egyptian Journal of Psychiatry - Start: 1978 Last year done: 1983, 1986, 1988-89 Ongoing: no
Hospital and Community Psychiatry (continued as Psychiatric Services) - Start: 1966 Last year done: 1994 Ongoing: no
Journal of Consulting and Clinical Psychology - Start: 1980 Last year done: 1995 Ongoing: no
Journal of Intellectual Disability Research - Start: 1992 Last year done: 2000 Ongoing: no
Journal of Mental Deficiency Research (continued as Journal of Intellectual Disability Research) - Start: 1958 Last year done: 1991 Ongoing: no
Journal of Mental Science (continued as British Journal of Psychiatry) - Start: 1948 Last year done: 1962 Ongoing: no
Journal of Nervous and Mental Disease - Start: 1948 Last year done: 1993 Ongoing: no
Korean Journal of Schizophrenia Research – Ongoing yes
Pakistan Journal of Clinical Psychiatry - Start: 1991 Last year done: Ongoing: no
Polish Journal of Psychiatry - Start: 1980 Last year done: 1994 Ongoing: no

Psychiatria Fennica - Start: 1970 Last year done: Ongoing: no
Psychiatric Services - Start: 1995 Last year done: Ongoing: no
Psychological Medicine - Start: 1970 Last year done: 1997 Ongoing: no
Psychosis – Start: 2009 Last year done 2015 Ongoing: yes
Psychosomatic Medicine - Start: 1971 Last year done: 1976, 1981, 1986, 1991 Ongoing: no
Schizophrenia Bulletin - Start: 1969 Last year done: 2008 Ongoing: yes
Schizophrenia Frontier – Ongoing: yes
Schizophrenia Research - Start: 1988 Last year done: 2008 Ongoing: yes
The following journals have been/are being searched and relevant trials are entered into the Register:
Zhurnal Nevropatologii I Psikhiatrii Imeni SS Korsakov - Start: 1980 Last year done: 1995 Ongoing: no

Additional Search Strategies
Citation Searching: This can be undertaken either electronically or manually. The methods by which reports are selected for citation searching are made explicit and reproducible.
Personal Contact: The methods by which individuals were selected for contact are made explicit and reproducible.
Pharmaceutical Industry: The methods by which companies are selected for contact and, in turn, how they select material for the review are made explicit and reproducible. It is important that pharmaceutical companies are not contacted directly but through the editorial base. CSG works to foster good relationships with pharmaceutical companies in an attempt to obtain the largest dataset possible. Individual approaches may lead to rejection, duplication of effort and the perception that CSG's efforts are ill-coordinated.
